# Current State of the Art and Next Generation of Materials for a Customized IntraOcular Lens according to a Patient-Specific Eye Power

**DOI:** 10.3390/polym15061590

**Published:** 2023-03-22

**Authors:** Martina Vacalebre, Renato Frison, Carmelo Corsaro, Fortunato Neri, Antonio Santoro, Sabrina Conoci, Elena Anastasi, Maria Cristina Curatolo, Enza Fazio

**Affiliations:** 1 Dipartimento di Scienze Matematiche ed Informatiche, Scienze Fisiche e Scienze della Terra (MIFT), Università di Messina, V.le Ferdinando Stagno d’Alcontres 31, 98166 Messina, Italy; 2 Optical Consultant SIFI SpA, 95025 Aci Sant’Antonio (CT), Italy; 3 Dipartimento di Scienze Chimiche, Biologiche, Farmacologiche ed Ambientali (CHIBIOFARAM), Università di Messina, V.le Ferdinando Stagno d’Alcontres 31, 98166 Messina, Italy; 4 Innovation and Medical Science, SIFI SpA, 95025 Aci Sant’Antonio (CT), Italy

**Keywords:** intraocular lens, GRadient INdex lens, acrylic materials, hydrogel, hydrophobicity, hydrophilicity, aberration, MTF

## Abstract

Intraocular lenses (IOLs) are commonly implanted after surgical removal of a cataractous lens. A variety of IOL materials are currently available, including collamer, hydrophobic acrylic, hydrophilic acrylic, PHEMA copolymer, polymethylmethacrylate (PMMA), and silicone. High-quality polymers with distinct physical and optical properties for IOL manufacturing and in line with the highest quality standards on the market have evolved to encompass medical needs. Each of them and their packaging show unique advantages and disadvantages. Here, we highlight the evolution of polymeric materials and mainly the current state of the art of the unique properties of some polymeric systems used for IOL design, identifying current limitations for future improvements. We investigate the characteristics of the next generation of IOL materials, which must satisfy biocompatibility requirements and have tuneable refractive index to create patient-specific eye power, preventing formation of posterior capsular opacification.

## 1. Introduction

An intraocular lens (IOL) is an artificial lens used to replace the eye’s natural crystalline lens when it is affected by cataract or other degenerative processes rendering it opaque [1]. The eye lens is structurally composed of 65% water and 35% proteins that, on aging, denature, reducing lens transparency. The increasing need of specific and personalized demands pushes the development of advanced IOL materials and design. Thanks to advances in ocular technology, there are now a wide range of premium intraocular lenses able to efficiently prevent adverse effects after IOL implantation and surgery, filter specific wavelength, and provide accommodation ability [2]. Injectable, shape memory, and adjustable IOLs are some of the innovative IOLs already present in the market [3]. In the first case, a full-size lens is obtained by injection of the lens material into the capsular bag of the eye as a fluid, followed by formation of a solid. Then, the lens is implanted through a 1.2 mm opening. An advantage of this technique is that, due to the formation of a full-size lens, it might overcome complications of conventional IOL implantation, namely decentration and posterior capsular opacification [4]. External stimuli (light, temperature, PH, etc.) can induce shape modification in suitable polymeric systems. For permanent modifications that can be restored by proper stimuli, we deal with the shape memory effect (SME) that applies to shape memory polymers (SMPs). IOLs made by SMPs can be reduced in size to require a tiny surgical incision, and then they re-acquire the initial size thanks to a change in environmental conditions [5]. 

Lenses constituted by photosensitive molecules (macromers) within a silicone matrix are known as light-adjustable lenses (LALs) [6]. If a specific region is irradiated with a 365 nm UV light, a polymerization reaction takes place, causing a precise photo-inducted concentration gradient that can modify the refractive index of the IOLs in post-surgery. In this field, the most innovative promises are RxSight’s (formerly Calhoun Vision) light-adjustable lens (RxLAL), approved by the Food and Drug Administration in 2017, and Perfect Lens femtosecond laser technology. Hence, their goal is modifying the refractive properties of an implanted IOL after surgery to accomplish a patient-specific customized device [7,8].

**Figure 1:**
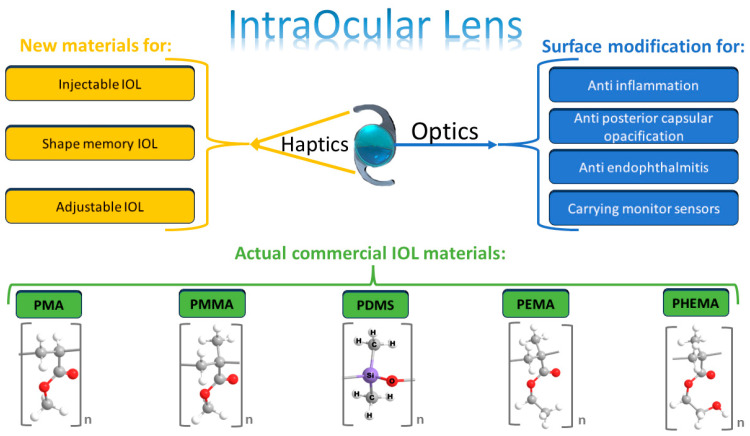
Scheme of commercial IOL indicating the main elements of an IOL: optics and haptics. The effects of IOL surface treatments and the main polymeric materials adopted in commercial IOL. Adapted from Ref. [2] under the terms of the Creative Commons Attribution (CC BY) license. Copyright © 2022 Luo, Wang, Chen, Xu, Yin and Yao.

IOLs are mainly composed of two elements: optics and haptics. Optics include the central area responsible for refraction, and haptics are the appendages from the centre optics that hold them in place. In cataract surgery, generally, two haptic designs of IOLs are used: single-piece and three-piece. Single-piece IOLs (also referred to as one-piece) are named as such due to both elements being composed of the same material (acrylic, silicone, or PMMA) [9]. However, by 2017, hydrophilic and hydrophobic acrylates accounted for 85% of the global IOL market share for IOL materials, while PMMA accounted for 12% and silicone a mere 3% of the market share. Acrylic materials can be rigid (PMMA) and foldable and made of hydrophobic acrylic materials and hydrophilic acrylics (see Figure 1). 

Nevertheless, materials continue to improve, becoming more pliable and biostable and giving IOL manufacturers greater freedom to address specific vision needs. IOLs have interesting features thanks to their material’s unique properties, such as refractive qualities, and aspheric designs capable of correcting presbyopia and astigmatism [10]. During surgery, every kind of IOL is centred with respect to the optical axis passing through the centre of the undilated pupil in order to achieve the best visual acuity [11]. 

In addition, corneal astigmatism can be corrected by means of toric IOLs that can improve distance vision, preventing use of glasses. In such a case, correct estimation of preoperative astigmatism is of fundamental importance to achieve beneficial outcomes. Together with good centration, these IOLs need proper rotational orientation [12]. They also exist as multifocal IOLs (either refractive or diffractive), providing improvements in both near and far visual acuity [13]. Refractive IOLs use multiple focal points whose concentrical regions have different dioptric power. Diffractive IOLs, on the other hand, possess multiple diffractive zones on their rear surface. As multifocal IOLs, bifocal IOLs show both near and far focus, while trifocal IOLs also comprise intermediate focus [10]. 

**Figure 2:**
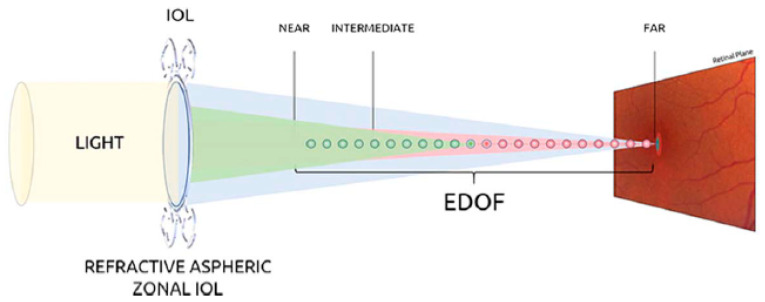
Basic principle of Mini Well and Mini Well Proxa EDOF-IOLs. Reprinted with permission from Ref. [15]. Copyright © 2023, Wolters Kluwer Health.

Recently, two new categories of IOLs, accommodative and extended depth of focus (EDOF) IOLs, are adopted. Accommodative IOLs provide dynamic refractive power, with contraction and relaxation of the ciliary muscles. EDOF IOLs provide extended far-distance focus through various techniques, which can improve intermediate vision [10]. For example, Mini Well and Mini Well Proxa (SIFI SpA, Catania, Italy) are hydrophilic EDOF IOLs that exploit an engineered wavefront surface to extend the depth of focus. They are made of a copolymer of HEMA (hydrophilic component) and EOEMA (hydrophobic component), which provides a good trade-off between elasticity and mechanical resistance. In addition, an optimized distribution of positive and negative spherical aberrations provides continuous focus from far to near distance [14]. Figure 2 reports the basic principle of Mini Well and Mini Well Proxa EDOF-IOLs, and some other details will be reported in the following sections. Another EDOF IOL, commercially available as IC-8 (AcuFocus, Irvine, CA, USA), is made of hydrophobic acrylic, with the central circular mask composed of polyvinylidene difluoride and carbon nanoparticles. The IC-8 IOL implements the pinhole effect to enlarge the range of vision [15]. Each of the engineered IOLs, differing in materials and design, offers unique advantages and disadvantages in view of specific applications.

In this mini-review, we report the main concepts regarding IOL technology, with a particular focus on the properties of their constituent materials. However, although the physical attributes of IOLs are often confused with the impact of their material, the latter affects function differently even within material categories. We should not assume that a given lens will behave in the same way as others in the same material category. In addition, well-designed IOL features have been determined in order to produce enhanced visual outcomes and improve quality of life of implanted patients and promote their social integration. 

Here, first, we will attempt to differentiate the physical characteristics of a lens from the effects of its material (i.e., the optical quality of pseudophakic eyes will depend not only on aberration control but also good media transparency and low light scattering). Then, recent new design of IOLs for correcting chromatic, spherical, and off-axis aberrations in the eye, as well as the advantages and limits of multifocal and GRIN (GRadient INdex—with variable refractive index)-based IOLs, will be presented. To this purpose, our Zemax simulation data are presented and described with a comparison between biconvex and GRIN lenses. 

## 2. IOLs Material Market

**Figure 3:**
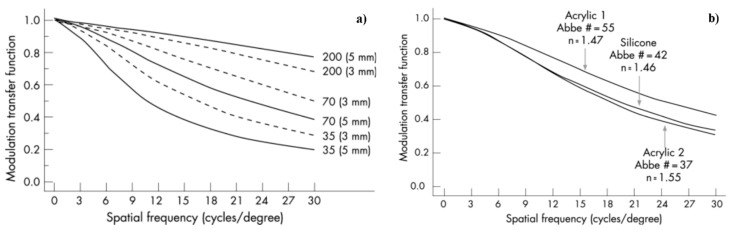
(**a**) Photopic polychromatic MTFs for an IOL with refractive index of 1.46 and Abbe numbers of 35, 70, and 200. Results for 3 mm and 5 mm pupil diameters are shown. (**b**) The MTFs trend for a silicone and two different acrylic IOLs. Reproduced from British Journal of Ophthalmology, Zhao, H.; Mainster, M.A., 91, 1225–1229, copyright 2007 with permission from BMJ Publishing Group Ltd. [20].

The earliest IOLs were made with thermal plastic, a material that improved dramatically over the years but still had inherent restrictions for microsurgery [16]. After decades of dominating the intraocular lens (IOL) material market, hydrophobic and hydrophilic acrylates may soon face competition from non-acrylate materials. Nowadays, the state of the art on acrylic materials offers the market improved plasticity and stability features by providing superior control during microsurgery. In general, the advantages of hydrophobic acrylate IOLs include a higher refractive index, which enables thinner IOLs, adhesive properties that provide good rotational stability, and a low rate of posterior capsule opacification (PCO). Alternatively, hydrophilic IOLs can be easily moulded into any desired shape and are flexible enough to enable injection through very small incisions. Whether hydrophobic or hydrophilic, opacification is a problem with all acrylate material lenses. In hydrophobic IOLs, this takes the form of “glistenings”, microvacuoles that result from fluid seeping into the empty spaces within the material’s polymer structure [17]. In hydrophilic IOLs, opacification is an outcome of calcification, which can result from defects in the material, and changes in the aqueous milieus may occur during intraocular surgery and in patients with conditions such as uveitis and diabetes [18]. Optical bench testing indicates glistenings have little effect on modulation transfer function (MTF) but significantly increase stray light [19]. MTF is a measurement of lens ability to transfer contrast at a particular resolution from object to image. MTF is a parameter expressing both resolution and contrast: as line spacing decreases (i.e., frequency increases) on the test target, it becomes increasingly difficult for the lens to efficiently transfer this decrease in contrast; as a result, MTF decreases (Figure 3a). Ultimately, IOLs with higher Abbe numbers (lower chromatic dispersion) have better optical performance and potential contrast sensitivity (magnitude of contrast sensitivity function is proportional to product of MTF and retinal–neural transfer function in Fourier space) as defined in Ref. [20]. Chromatic dispersion affects the overall pseudophakic performance of spherical-aberration-correcting IOLs. In Figure 3b is shown the MTFs trend for a silicone and two acrylic IOLs. The IOLs have different radii of curvature of their anterior surfaces in order to ensure zero spherical aberration for their optic materials.

Cross-linked polyisobutylene (xPIB) is today under investigation as an innovative IOL material. It has a high refractive index (1.52) and is highly elastic, meaning it could be used for large-optic IOLs implantable through a sub-2.0 mm incision. It also induces less chromatic aberration. Moreover, its molecular structure ensures xPIB to be not susceptible to glistening or calcification [21]. We outline that resistance to glistening formation differs depending on the hydrophobic acrylic copolymer composition of the IOL material.

The hygroscopic nature of a polymer changes depending on the temperature and ionic strength of the surrounding solution [22]. As water diffuses into the polymer due to equilibrium driving forces, discreetly visible vacuoles can develop [23]. However, why some IOLs are more prone to form glistenings than others is poorly disclosed in the literature. Mao et al. [24] used molecular dynamics simulation methods to conduct in-depth research on the molecular mechanism of the glistening formation of IOLs, aiming to provide a possible molecular mechanism of glistening. Furthermore, based on this molecular mechanism, Mao et al. [24] proposed a novel strategy of a glistening-free material based on a composite design method to reasonably copolymerize several types of molecules or functional groups so that the glistening phenomenon can be effectively eliminated. Some characteristics regarding IOL-based materials are reported in Table 1, while further details will be described in the next sections. Among the parameters reported in Table 1, we defined the Abbe number as an indicator of the optical material’s chromatic dispersion: V_d_ = (n_d_ – 1)/(n_F_ – n_C_), where n_d_, n_F_, n_C_ are material’s refractive index at 587, 486, and 656 nm, respectively. Generally, materials with lower chromatic dispersion have larger Abbe numbers. Refractometers are often used to measure Abbe numbers, which range from 35 to 60 for contemporary IOL materials [25,26].

### 2.1. Silicone

Silicone is widely used in IOLs applications, is a nontoxic and biologically inert polymer, structurally constituted by monomers of dimethylsiloxane (DMS), and hydrophobic, as demonstrated by contact angle measurements, which evidenced a contact angle using water of 99°. Such polymer, without further functionalization, shows several disadvantages to be used as IOL: (i) it is slippery when wet, so it must be handled dry if folder and holder forceps are employed for implantation; (ii) the rather low refracting index (between 1.41 and 1.47) implies rather thick optics and, therefore, a larger incision (about 3.2 mm or larger) during surgery (see Table 1); (iii) it is prone to PCO. To improve its performance and overcome possible issues in IOL applications, polydimethylsiloxane (PDMS) structure is normally functionalized. The shape of IOL made by PMDS bearing photoreactive coumarin groups can undertake reversible postoperative adjustment by using UV light. Specifically, coumarin groups enable crosslinking reaction of PDMS upon irradiation with wavelength above 300 nm by forming a cyclobutene ring. Such reaction can be reversibly cleaved by irradiating the polymer with wavelengths below 290 nm [27], so it would be possible to adjust the shape of the lens after implantation and correct eventual errors that can occur during such procedure.

In recent years, the design of injectors for 3-piece silicone lenses enabled more accurate handling of IOLs [28,29]. The sharp surgical opening to place a silicone IOL inside the anterior chamber is still an unsolved issue. Moreover, silicone lenses can promote bacterial adhesion and thus postoperative infections. Therefore, inflammatory reactions in the eye and capsular opacification (both posterior and anterior) are favoured with respect to hydrophobic acrylic IOLs, as discussed below. Furthermore, use of silicone IOLs has been correlated with calcification phenomena in eyes with asteroid hyalosis. Finally, probably due to the relatively low refractive index, silicone IOLs better tolerate pseudophakic dysphotopsia compared to many acrylic IOLs [30]. In summary, silicone-based IOLs are heat-resistant, autoclavable, mouldable, compressible, highly transparent to visual light, and very flexible, providing good tensile and tear strength. Otherwise, they are slippery, have a lower refractive index than acrylic lenses, and show high adherence to silicone oil after vitreoretinal surgery.

### 2.2. Acrylic Materials for IOLs

Most lens implants are today performed with an acrylic material. They are today’s most popular choice in part because they are easy to fold and ideally suited for microsurgery and also because IOLs made from polyacrylic material are associated with a significantly reduced degree of PCO with respect to PMMA and silicone ones. Ni Li et al. [31] showed that AcrySof (hydrophobic acrylic material with higher fibronectin bio-adhesion properties and a sharp optic edge) and sharp-edged silicone IOLs are similarly effective in inhibition of PCO after cataract surgery. Companies such as Benz Research and Development provide high-quality raw materials, such as HEMA-EOEMA, at 99.9% purity for the IOL industry. In Table 2 are described its main characteristics in comparison to those of other acrylic materials.

PMMA is the material used in Sir Harold Ridley’s original intraocular lens. Until the last decade, PMMA has enjoyed a reputation as a reliable and high-quality optics material. It has a refractive index of 1.49 and usual optic diameter 5–7 mm. Nevertheless, its main downsides are (1) larger incisions are necessary because PMMA is not foldable and conducive to micro procedures as newer materials; (2) very severe glistening developed by some injection-moulded PMMA lenses; (3) snowflake degeneration is a slowly progressive opacification of PMMA IOL, which may occur 10 years or later after implantation. For this reason, hybrid acrylic polymers provide IOLs that combine the advantages of both PMMA and silicone, i.e., suitable refractive index and tuneable foldability [32]. Recently, PMMA surface functionalization with either hydrophilic or hydrophobic polymers has been created in order to improve its performance. For example, 2-hydroxyethyl methacrylate (HEMA) was immobilized on hydrophobic polymethyl methacrylate intraocular lens to increase the hydrophilicity degree of IOLs. In addition, the HEMA2-g-PMMA IOL group suppresses proliferation of cells [33].

**Table 2:** IOL-acrylic-based materials. Data from Ref. [34].

Materials	Refractive Index	Water Content	Flexibility
MMA	1.49	<1%	rigid
PEA/PEMA	1.55	<1%	foldable
EA/EMA/TFEMA	1.47	<1%	foldable
HEMA/MMA	1.47	20%	foldable
HEXMA/HEMA	1.47	18%	foldable
HEMA	1.44	38%	foldable

#### 2.2.1. Hydrophobic Acrylic Materials

In the field of hydrophobic acrylic materials for IOLs, the most used polymers are made of esters of poly(meth)acrylic acid, mainly poly(2-phenylethylmethacrilate) (poly PEMA), poly ethylmethacrilate (poly EMA), and poly(2,2,2-trifluoroethyl methacrylate) (poly TFEMA). They absorb less than 1% water and remain foldable even when they are totally dry. The refractive index of such materials is relatively high, reaching 1.47, and even higher when the polymeric esters contain aromatic groups as poly(PEMA) (~1.55, AcrySof® by Alcon (Fort Worth, TX, USA)). The glass transition temperature (T_g_) of these polymers falls around room temperature; at lower temperature, the materials become rigid; for this reason, they require specific cooling tools to be processed. The first foldable IOLs system made by hydrophobic methacrylate was prepared in 1993 by co-polymerization of phenylethyl acrylate (PEA) and phenylethyl methacrylate (PEMA) cross-linked with butane diacrylate [35]. It immediately appeared as a good candidate for IOLs manufacturing and recent studies confirmed the first impressions. A study carried out in 2008 evidenced that the co-polymer made by PEA and PEMA monomers takes advantage of the high refractive index value (1.55) of PEMA to make thinner optic and unfold slower than silicone lenses, providing greater control during implantation. The “softness” can be tuned by varying the ratio of the two monomers in the final co-polymer. In specific, T_g_ values for the poly (phenylethyl) acrylate (PPEA) and poly (phenyl ethyl methacrylate) (PPEMA) homopolymer are 2 °C and 38 °C, respectively, and it was demonstrated that the T_g_ of the co-polymer can assume the values of 23, 17, and 10 °C by maintaining 27:73, 48:52, 70:30 PEA:PEMA ratio, respectively [36]. As reported in patent WO2010109043A1, another commercially available co-polymer used for IOLs application is constituted by ethyl acrylate, ethyl methacrylate, and 2,2,2-trifluoroethyl methacrylate (poly(EA/EMA/TFEMA)), commercially Allergan Clariflex^®^ (Allergan, Inc., Irvine, CA, USA). previously Abbott Medical Optics (AMO) and now AbbVie Inc., North Chicago, IL, USA), with a water content similar to the PEA-PEMA co-polymer (around 1%) but with a refractive index of 1.47 [37].

#### 2.2.2. Hydrophilic Acrylic Materials

Use of hydrophilic acrylic materials in the manufacture of IOLs started for the same reasons as hydrophobic. Such materials are formed of poly(2-hydroxyethyl methacrylate) (PHEMA) hydrogels with hydration of 20% or higher in water. The IOLs constituted by such homopolymer results foldable and with contact angles ranging from 59 to 69°. Unfortunately, the low value of refractive index of this material (1.44) is an obstacle when used to make thin IOLs (Alcon HydroSof^®^ is an example of such IOLs, with a degree of hydration of 38% and refractive index of 1.41). For such reason, the HEMA monomer is often co-polymerized with other materials with a higher value of refractive index, as in the case of ORC MemoryLens^®^ (ORC Technologies, Inc., Azusa, CA, USA), as reported in patent WO2010109043A1. HEMA co-polymerizes with MMA to obtain a flexible IOLs with 1.47 and a degree of hydration of 20% [37]. Another interesting example concerns the case of Storz Hydroview^®^ (Storz Ophthalmics, Heidelberg, Germany), in which HEMA is co-polymerized with 6-hydroxyhexyl methacrylate (HEXMA) monomer, with a refractive index of 1.47 and hydration of 18%. Other examples have been shown by HEMA and HEXMA co-polymerized with different cross-linking agents. 

These kinds of polymers can absorb water from 10–20% of their total weight up to thousands of times their weight, forming hydrogels [38,39]. A crosslinked polymer thus obtained constitutes a sort of net to “trap” water molecules. The amount of absorbed water can be conveniently chosen by varying the amount of cross-linking agent used in the preparation or by introducing from small to moderate amount of hydrophobic monomers during the synthetic step. For IOL applications, the water content of hydrophilic acrylic polymers is in the range of 18–38%. Such water content confers to acrylic lenses excellent biocompatibility, high softness degree, and good compressibility. These materials show a water contact angle lower than 50° and a refractive index around 1.43. Although after surgery they show low probability for induction of photopsias, the PCO rate is somewhat high due to calcium deposits and system viscoelasticity. Hydrophilic acrylic IOLs, mainly constituted by 2-hydroxyethyl methacrylate (HEMA) and 6-hydroxyhexyl methacrylate (HEXMA), are flexible for water content in the range of 18–38% and turn out to be rigid and unfoldable in dry conditions [40]. To overcome this limitation, phakic IOLs combine HEMA with collagen (collamer lenses) [41]. Hydrophilic IO lenses fold and unfold faster than the hydrophobic counterparts and result more controllable than silicone lenses [42].

As briefly discussed for PDMS, with hydrophilic acrylic materials, it is also possible to modify the bulk of the IOLs raw material by including molecules to achieve a programmed task. One example is represented by the copolymer of which is made the MINI WELL lens (SIFI SpA, Catania, Italy) [43] based on a hydrophilic monomer, the 2-hydroxyethylmethacrylate (2-HEMA), and a more hydrophobic ethoxy-ethylmethacrylate (EOEMA) one. The ratio of the two constituents could range from 40 to 90% in weight of the hydrophilic component depending on the desired properties that the IOLs device should possess. The refractive index of such lens falls between 1.4 and 1.5 depending on the ratio of monomers used and on the water content (which is from about 10 to 38% of the total weight of the copolymer). Regarding water content, the refractive index value progressively decreases by increasing the water content. During polymerization of the bulk material, an ultraviolet absorbing compound can be incorporated inside the polymer matrix, so it can resist the possible extraction under physiologic conditions. Generally, the concentration of such agent is between 0.01 and about 1% in weight of the copolymer, enabling to provide the desired UV-absorbing properties without modification of lens transparency. The monomers used for the bulk polymer confer to the MINI WELL IOL other advantages: in particular, it is an example of one-piece IOL, so there are no junctions between optics and haptics parts because they are made of the same material. The properties achieved by the polymer well match both the desired characteristics for the optical and haptical part. In addition, the developed IOLs are foldable and thus can be inserted into the eye through a “small” incision. For more details, see patent WO1999056672A.

#### 2.2.3. Innovative Hybrid and Flexible Polymeric Materials

Nowadays, technology helps to increase the efficiency of IOLs mainly made of hybrid materials with a high refractive index for flexible handling and a high surface quality. With a water ratio less than 5%, these hydrophobic-hybrid materials stand out from many of the dry-stored hydrophobic IOLs. Here, further information about hydrogels IOLs is reported. Some of them are constituted by a cross-linked polymer, namely the poly-hydroxyethyl methacrylate or poly-HEMA, showing rigidity in dry conditions but becoming a foldable gel when hydrated. Today, the demand of foldable lenses allowed the design of a wide range of different flexible hydrophobic IOLs. As mentioned, these materials consist of a copolymer mixing different monomers, such as methymethacrylate (MMA), ethylmethacrylate (EMA), 2-hydroxyethyl methacrylate (HEMA), etc. The chemico-physical properties of such materials can be conveniently tuned by varying the composition ratio of the monomers and the kind of cross-linking that in turn defines the flexibility of the side chains. For instance, PMMA is used to confer more rigidity to the system, whereas PEMA to furnish a certain elasticity. Finally, the presence of the OH group in poly-HEMA enhances the system hygroscopic properties [44].

One important approach adopted for PCO prevention consists of surface modification of IOLs. A thin coating of the surface does not change the physical properties of the bulk (refractive index, foldability, Young’s modulus), but it can be used to endow some interesting features to the whole device, improving biocompatibility, reducing PCO occurrence, and so on. Depending on the characteristics that the IOLs system has to assume, the coating can be hydrophilic or hydrophobic. To create a hydrophobic coating, perfluorinated precursors are usually used [45,46]. Fluorinated coating makes the surface of the lenses inert toward cells, so the capsular opacification due to growth in the epithelial cell is noticeably reduced. The above-mentioned feature expressed by the hydrophobic coating has effect also in lenses made by poly(MMA). Modified lenses become also more slippery, thus easier to implant through smaller incisions. On the other hand, to make highly hydrophilic coating, poly(ethylene oxide) (PEO) is often used due to its non-toxic, non-immunogenic, and non-antigenic properties [47]. The high mobility of the hydrated PEO segments avoids deposition of the proteins on the lens surface thanks to steric repulsion, thus preventing formation of an extracellular matrix and adhering of the epithelial cells. For the properties that such coatings confer, poly(MMA) and hydrophobic acrylic IOLs are often coated with PEO [47,48]. It was also demonstrated that linear PEO grafted on the surface of poly(HEMA-*co*-MMA) lens has better performance in IOLs applications compared to the brush type polymer due to partial lens opacification effect that the latter showed. Moreover, cell repulsion efficiency can be improved by increasing the length of the polymer chain grafted on the lens without affecting lens transparency [49,50]. Similar to PEO, heparinization of the surface of IOLs also provides anti-adhesive protein feature as well as inhibition of cell growing. The mechanism is the same of PEO, the hydrophilic medium on the surface traps the water molecules and develops a hydrated surface that protects the bulk lens from cells growing [46,51,52].

### 2.3. Advanced Materials for Injectable, Shape Memory, and Adjustable IOL

Innovative materials such as hydrogels and biodegradable polymers, with the aid of nano-technology, are being applied to tissue engineering with the aim to reproduce the physico-chemical properties of eye tissues and deliver and also control the delivery or release of active biomolecules [2]. Drug-eluting IOLs can be used from one side to inhibit infections and other injuries of the eye tissues (i.e., avoiding opacification), and, from the other side, their stored drugs can be released for treatment of some specific pathologies [53]. However, implantation procedures for such IOLs need fine abilities. In fact, inappropriate handling can damage the IOL system or provoke undesired effects during injection. Hence, the performance of IOLs injector systems should be carefully evaluated considering many parameters, such as the occurrence of anomalous leading haptic configuration, trapped trailing haptic, optic–haptic adhesion, and IOL attachment to the plunger [54]. The most used injector cartridge design is illustrated in patent US4681102 and includes a split longitudinally hinged cartridge. Similar designs are illustrated in patents US5494484, US5499987, US5616148, and US5620450. Viscoelastic lens materials, such as soft acrylics, are temperature-sensitive and roll or fold more easily at higher temperature. None of the prior art cartridges contain a feature that enables heating of the cartridge so as to warm the lens during insertion. Hence, a need continues to exist for a cartridge containing a heat retention additive that enables heating of the cartridge so as to warm the lens during insertion. Such additives can include a biocompatible material having high heat retention, for example, powdered gold (see patent US6398789B1). Ejection tests are carried out in vitro, measuring the resistance force throughout the entire delivery process. The effect of IOL material, haptic design, IOL thickest area, and ophthalmic viscosurgical device (OVD) have been studied by ejecting several IOLs with syringe-type injectors of different sizes, 3.0, 2.2, and 1.8 mm [55]. It has been observed that plate hydrophilic IOLs present the lowest resistance forces; hydrated C-loop hydrophobic IOLs present higher forces and the C-loop hydrophobic IOL in dry conditions presents the highest resistance forces. Therefore, only the combination IOL and specific injector lead to a reproducible safe result.

**Figure 4:**
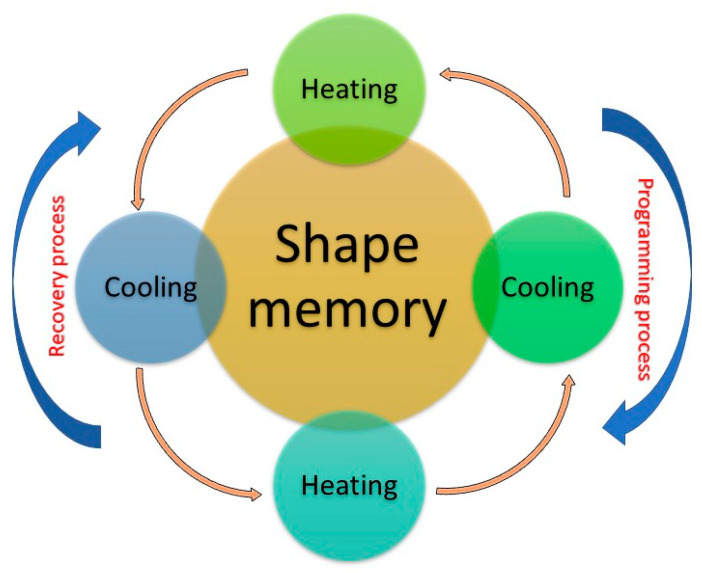
Representative scheme of SMP cycle. The material can be deformed when cold but returns to its pre-deformed (“remembered”) shape when heated.

Shape-memory polymers (SMP) are a class of smart materials that can be tailored to have significant mechanical property changes in response to a given stimulus (see Figure 4), inducing internal stress: in turn, energy will be released, causing the molecular chain to possess mobility again [56]. The polymer then returns to the original shape.

**Figure 5:**
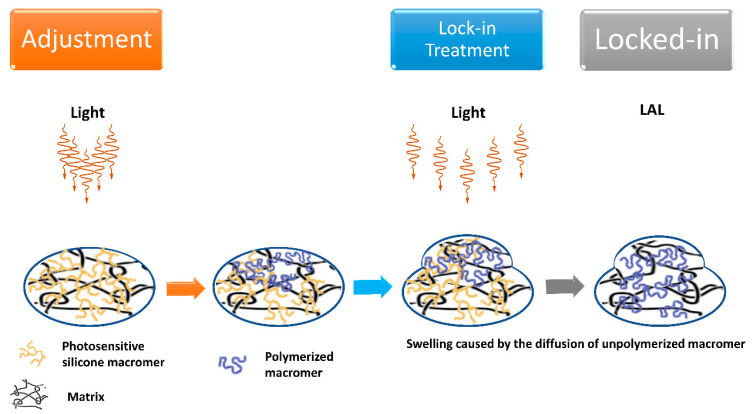
Scheme for new technology for making postop IOL adjustments.

Extensive use of SMPs in minimally invasive surgery can be attributed to their capability to retrieve the proper shape under an external stimulus after being deformed and to their great adaptableness to varying environmental conditions [57]. Today, SMPs can be realized with the wanted mechanical properties, such as modulus and glass transition temperatures (T_g_), and optical properties, including transparency and the refractive being close to that of the human natural lens (usually in the range 1.386–1.406), mainly by varying the cross-linker weight percentage and the ratios of each monomer in the co-polymer. In addition, their high biocompatibility leads to design of personalized treatments of specific ophthalmic diseases [58,59]. Application of stimuli such as heat or UV light enables modifying the curvature radius of both the anterior and posterior surfaces of some IOLs, tuning their central and/or paracentral power [60]. IOLs made by SMPs can have a refractive index of IOLs higher than 1.45, a T_g_ falling in the range 15–40 °C, usually show minimum or no glistening, and a value close to 100% of transmissivity of light in the visible region of the spectrum. SMP IOL can be created by using different ratios of tertbutyl acrylate (tBA), isobutyl acrylate, and poly(ethylene glycol)dimethacrylate (PEGDMA) 1000; for instance, it can be realized by 50% of tBA, 28% of isobutyl acrylate, and 22% of PEGDMA 1000, or by a 22% of tBA and 78% of PEGDMA 1000 or by 65% of tBA, 13% of butyl acrylate, and 22% of PEGDMA 1000. Although there could be different alternatives, a typical SMP IOL includes tBA and one or more PEGDMA monomers. Then, it can optionally include one or more UV-blockers and polymerization initiators, as well as n-butyl acrylate (nBA) and 2-hydroxy-3-phenoxypropyl acrylate (HPPA) [61]. In Figure 5 is shown a scheme for a new technology used for making postop IOL adjustments. The light adjustments are completed with a specific wavelength of UV light (365 nm). Each light treatment lasts approximately 20 to 30 s depending on the type of treatment, while the final lock-in adjustment treatment is longer. Once the lens finds a stable place in the eye, a few weeks after surgery, the patient is placed in front of the light delivery device for adjustments and the final lock-in.

Recently, shape memory polymer composites (SMPC) are an emerging class of insolent structural substances that can be inelastically abnormal and regain their standard shape by an external environmental change [62]. Light-adjustable lens (LAL) is the first and only premium IOL that can be fine-tuned and adjusted after insertion. Thanks to the LAL, you can customize your vision after cataract surgery. With other IOLs, you cannot adjust the lens power after your procedure if unhappy with the quality of your vision. In such a situation, the next best option is to have a corneal-based refractive surgery, such as LASIK, after cataract surgery [63]. The LAL system is constituted by the lens, insertion device, and light delivery device. The LAL is a photo-reactive 6 mm biconvex silicone lens showing ultraviolet absorption abilities with a rounded-edge anterior surface and a squared-edge posterior surface. The haptic moiety with 13 mm of total diameter is made of polymethylmethacrylate. During surgery, the lens is inserted into the capsular bag through a small 2.8 mm incision. After implantation, the lens is irradiated, causing photopolymerization of the irradiated part. Light adjustability of the lens is possible thanks to the gradient of monomers concentration created after the photo-polymerization. In detail, macromers that are not directly irradiated move into the exposed area following the concentration gradient and cause precise shape and power change. When the desired refraction index is reached, the whole system is posed under irradiation to lock the position of macromers through polymerization [64,65]. The LAL can be optimal for those who do not need multifocal lens but still want a premium lens, similarly for those who do not like to risk glare and dysphotopsias and those who desire precision monovision or precise distance vision with reader glasses. Today, the only LAL on the market approved by the FDA is RxSight’s RxLAL, a three-piece foldable silicone monofocal LAL implanted by means of a 2.8 mm corneal incision with an exclusive injector [66]. Its total diameter is 13 mm with a 6 mm optic, squared posterior optic edges, round anterior edges, and blue poly(methyl methacrylate) modified-C haptics with posterior optic-haptic 10-degree angulation. This LAL IOL exists in powers from +10 to +30 D, in 1-D increments from +10 to +15 D and +25 to +30 D, and in 0.5-D increments from +16 to +24 D [67]. Ultimately, the LAL has demonstrated to be a safe, accurate, and reliable method of postoperative, non-surgical correction of residual sphero-cylindrical refractive error. Clinical results have been positive, with the largest series (122 eyes) showing 97% of patients within 0.25 D of attempted spherical equivalent and 100% uncorrected vision 20/25 or better. However, LAL should not be advocated in the following situations: (1) pre-existing macular disease; (2) prior history of herpes eye infection; (3) intake of medications that increase sensitivity to ultraviolet (UV) light; (4) intake of retino-toxic medications; (5) patients with nystagmus.

### 2.4. Variable Refractive Index Materials for IOLs

In the design of lenses for ophthalmic use, a salient aspect has currently been overlooked due to some aberrations, which, however, cannot be correctly treated using a single material. As described in the previous sections, improved physico-chemical properties and functions are obtained by adding extra polymers or by surface modification. Innovations of primary importance today are creation of ophthalmic optics by combining several materials with different refractive indexes. The idea stems from well-established creation of achromatic lenses that correct chromatic aberration and improve image quality by juxtaposing two (or more) materials with different refractive index and Abbe number (remember that the V-number is an approximate measure of the material’s dispersion (change in refractive index versus wavelength). Fernandez and Artal showed that achromatic IOLs made with two different materials, such as acrylic and silicone, offer advantages in terms of improving the patient’s visual quality [68]. The authors demonstrated, by running numerical simulations, that hybrid IOLs made by a low dispersion material (with refractive index 1.47 and Abbe number 55) and a more dispersive material (with refractive index 1.55 and Abbe number 37) have the potential for near perfect aberration correction, avoiding use of diffractive surfaces [68]. 

Another class of materials consists of materials known by the acronym GRIN (GRadient INdex—with variable refractive index). Ophthalmic lenses could be made with such materials that make greater use of the variation in the refractive index rather than the curvature to optimally direct the light onto the retina [69,70]. A traditional lens designer can vary the curvatures, the thickness, and the refractive index of the material. In addition, a GRIN lens designer can also use index gradients to simplify or enhance the optical system. For example, axial gradients, where the index of refraction varies along the optical axis, can perform the same functions as an aspheric surface in a traditional lens. A traditional lens design that requires an aspheric surface can be replaced with a spherical surface that is substantially easier to form and polish if an axial gradient is introduced. Even more useful are radial gradient lenses, where the index of refraction is a function of the distance from the centre of the lens. Radial gradients can add focusing power and control specific aberrations. Thus, the main advantage of GRIN-based IOLs is that they would be much more anatomically similar to the crystalline lens (which has a rather complex layered structure) than the current technology with single material [71,72,73].

Use of GRIN materials results in possible construction of uniquely shaped lenses that lack the typical aberrations present in conventional spherical lenses. An excellent example of a gradient index optical system is the human eye [74]. The refractive index of the eye lens varies from 1.406 in the centre to 1.386 in the outer layers, allowing humans to see images with both good resolution and low aberration. The refractive index of the vitreous humour within the eye is typically around 1.3. Therefore, by precisely varying the refractive index of the surface, gradient index lenses are able to continuously bend light within the lens until the light rays focus on the focal point behind the lens. In detail, as light passes from the front of the human eye lens to the back, light rays are refracted by varying degrees. It is a very efficient means of controlling the pathway of light without relying on complicated optics and one that we attempted to mimic. This contrasts with conventional spherical lenses that bend light only twice: when the waves meet the surface of the lens and when they exit the back of the lens. GRIN lenses are of course subject to the Abbe–Rayleigh diffraction limit, so they do not allow to observe objects with dimension smaller than half wavelength of light. However, to overcome this problem, some scientists proposed to use metamaterials to alter in a controlled way the morphological distribution of lens materials with the aim to manipulate the electromagnetic field of the propagating light. These are known as super GRIN lenses, in which the electromagnetic beam is compressed in order to propagate within the lens, achieving fine focus better than conventional GRIN lenses [75].

Current-generation intraocular replacement lenses, such as those adopted to treat cataracts made from a homogeneous single material, use their shape to focus light to a precise prescription, similar to glasses and contacts. Such lenses never achieve the same performance of natural lenses because they lack the ability to incrementally change the refraction of light. This single-refraction replacement lens can create aberrations and other unwanted optical effects. These latter effects are strongly reduced in MIOLs (multifocal intraocular lenses), characterized by variable refractive power associated to the anterior and/or posterior surface shape. GRIN-MIOLs’ main advantage is determined by the gradient optics obtained by changing the refractive index in the inner structure of the IOL. In this way, optical side effects are diminished, also improving functional results [69]. Nevertheless, gradient IOLs show some advantages compared to MIOL design, such as mimicking normal physiology; a smooth optical surface that allows to decrease the mechanical damage to the lens optic during implantation; postoperative better retinal image quality; and functional vision achieved at near and intermediate distances and under varying light conditions (photopic, mesopic, and scotopic).

GRIN technology is still evolving today. There are several quite different optical fabrication methods for GRIN lenses whose choice strongly influences the lens performance [76,77,78,79]. Specifically, the technological basis for creating nanolayered polymer GRIN optics lies in the fabrication of polymer films with a tailored refractive index. When two polymer materials with a sufficient difference in refractive index are arranged in alternating layers, the resulting layered material has a refractive index modulation whose period corresponds to layer thicknesses [80]. Recently, the direct laser writing, induced by a femtosecond laser, is largely used to locally change the refractive index of several ophthalmic materials, such as hydrogels [77]. For instance, Sola and Cases processed acrylic IOLs (a 320 µm thick hydrophobic UV-photo-reactive polybenzylmethacrylate polymer) with 250 fs laser pulses at 520 nm wavelength and 63 MHz repetition rate to inscribe low damage diffraction gratings [81]. Indeed, they were able to create linear periodic patterns both on the surface and inside the acrylic substrate by varying the parameters for the micromachining process, such as inter-line spacing (10–40 µm), scanning speed (0.25–1 mm/s), and pulse energy (1–2 nJ), achieving a refractive index change between 2.8 × 10^−3^ and 4.00 × 10^−3^. Recently, there have been successful attempts to locally change the refractive index of an existing IOL by using femto-second laser pulses. This technique is termed intra-tissue refractive index shaping (IRIS) [82]. The IRIS technique is a novel, non-ablative form of vision correction by which femtosecond laser pulses are tightly focused into ocular tissues to induce localized refractive index (RI) change via nonlinear absorption. By regulating the energy of the femtosecond laser pulse with an acousto-optical modulator at the speed of about 1 MHz, the laser beam is focused on a small region of the IOL. Other than the changes in material provoked by the heat, use of a suitable wavelength allows altering the polarity of some functional groups, modifying the wettability properties of the polymeric surface and indeed the corresponding refractive characteristics [83].

### 2.5. Zemax Simulation Data: Comparison between Two Lenses (One Biconvex and One GRIN)

To further clarify what is reported in the previous paragraph, the comparison between two lenses (one biconvex and one GRIN) with the same diameter (6 mm) and focal length (12 mm) is shown. The biconvex lens is for simplicity formed by two surfaces with the same curvature, while, in the GRIN lens, the back and front surfaces are perfectly flat. In this second case, the optical power is conferred on the lens by a radial variation in the refractive index of the medium, which, despite the absence of curvature, manages to “curve the rays” in such a way as to practically cancel the spherical aberration. To highlight this latter concept, both the spot where the rays converge on the focal plane (spot diagram) and the ray fan plot have been reproduced in the two cases analysed using the Zemax software; for simplicity, only monochromatic light (550 nm) is considered. 

In Zemax and other optical design codes, ray fan plots are determined by tracing many rays from a single object point through many selected locations in the entrance pupil to the image plane, while spot diagrams are maps of where rays intersect the image plane after passing through the pupil with a chosen grid pattern. Ultimately, a spot diagram is a graph showing where the rays coming from a point object converge on the image surface: the smaller the spot size on the image surface, the better the image quality. In our examples, the object point is placed on axis and at an infinity distance from the lens aperture; therefore, the rays coming from it are almost parallel to the optical axis.

**Figure 6:**
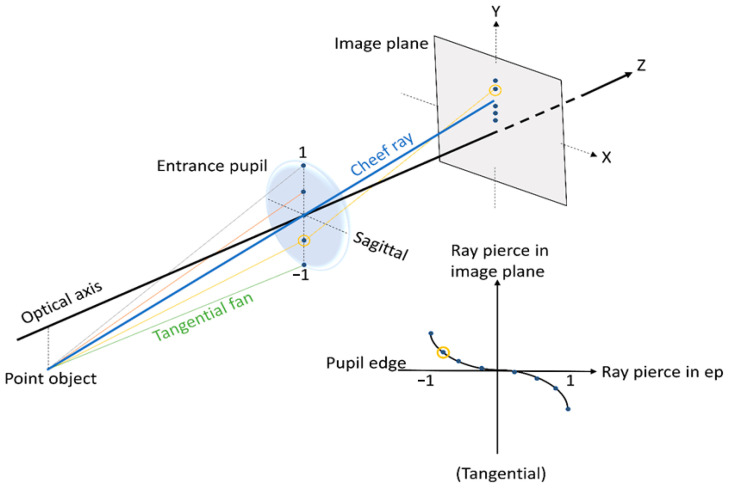
Ray fan plot geometry.

Moreover, rays represent the direction of wavefront propagation. Therefore, rays point in the direction of the wavefront surface normal and can be calculated as the wavefront gradient. The “transverse ray aberration” (*TRA*) is the distance, orthogonal to the optical axis, between a paraxial ray and its corresponding real ray (i.e., the transverse distance between ideal and real ray locations). The *TRA* can be calculated as a derivative of the wavefront:

$$TRA\left(y\right)=-\left(\frac{R}{nr}\right)\frac{\partial W}{\partial y}$$

where *R* is the radius of curvature of reference sphere; *r* is the exit pupil height; *n* the index of refraction in image space; *W* the wavefront aberration function (OPD); and *y* the meridional-plane (vertical) coordinate in exit pupil. “Ray fans” plot the ray aberration vs normalized pupil coordinate in tangential and sagittal planes. In Figure 6 is shown a scheme of the ray fan plot geometry [84].

For our biconvex and GRIN lenses, the simulation procedure was implemented as follows. First, the homogeneous material has been defined by assigning the refractive index n and Abbe number V values. Therefore, a biconvex lens made of a copolymer of HEMA (hydrophilic component) and EOEMA (hydrophobic component) characterized by *n* = 1.46 and *V* = 47 is simulated. Instead, the punctual distribution of the refractive index n in the medium was analysed to simulate the GRIN lens. We outline that the ray trace through a gradient index medium requires iteration to determine the point of intersection of the ray with the surface following the gradient index surface. 

**Figure 7:**
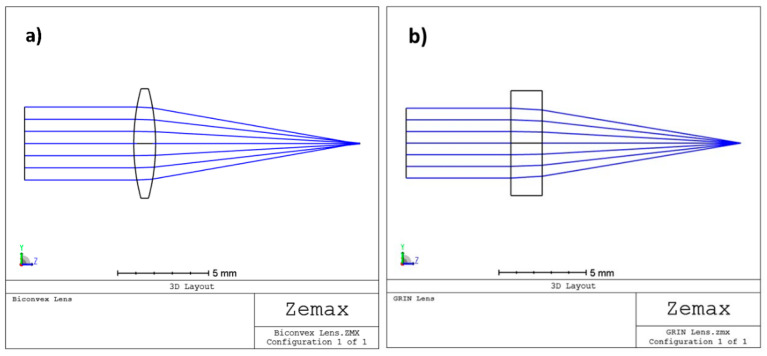
Layouts for (**a**) biconvex lens; (**b**) GRIN lens.

**Figure 8:**
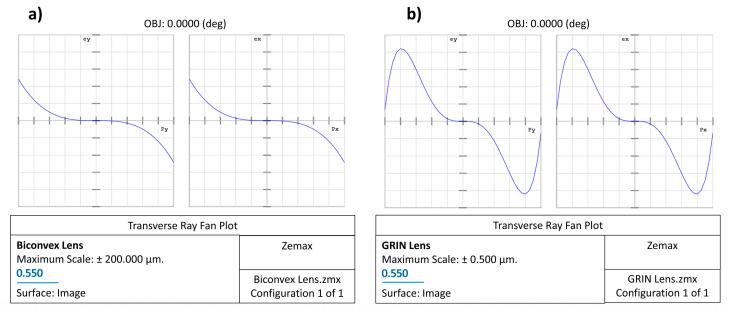
Ray fan plot for (**a**) biconvex lens; (**b**) GRIN lens.

**Figure 9:**
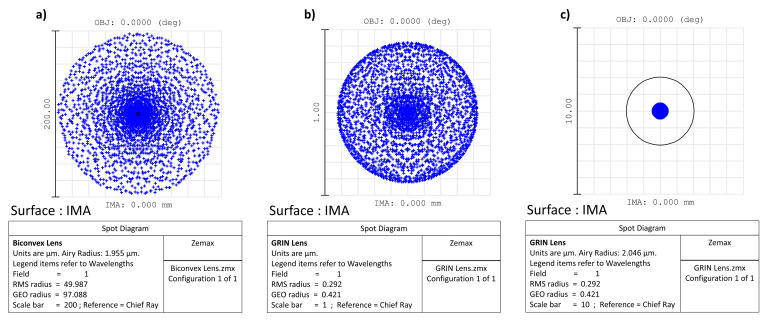
The diameter of the spot projected onto the image plane for (**a**) biconvex lens; (**b**) GRIN lens; (**c**) airy disc for the GRIN lens.

In our case, a “Gradient 3” was used for the simulated GRIN lens: this surface type has the same shape as the standard surface with medium whose index of refraction is described by n = n_0_ + n_r2_·r^2^ + n_r4_·r^4^ + n_r6_·r^6^ + n_z1_·z + n_z2_·z^2^ + n_z3_·z^3^, where r^2^ = x^2^ + y^2^. Seven parameters are required: the base index n_0_ and the remaining six coefficients of the previous equation. In this example, for simplicity, we set n_0_ = 1.65 and only the radial terms have been optimized to obtain the same focal length of the previous biconvex lens and minimize the whole spherical aberration obtaining in the end: n_r2_ = −2.3507 × 10^−2^, n_r4_ = 4.844 × 10^−5^, and n_r6_ = 3.7549 × 10^−7^. In Figure 7 are shown the layouts for the biconvex and GRIN lenses, respectively. Figure 7a shows that the incoming rays apparently focus on a small spot on the image surface for both lenses; however, the biconvex lens has a significant amount of residual spherical aberration. This is most evident looking to the ray fan plot shown in Figure 8a. Consequently, the diameter of the spot projected onto the plane of best focus is also very large (Figure 9a).

Unlike the case of the biconvex lens, for the GRIN lens, the parallel incoming rays converge to a very small spot (Figure 7b). Therefore, even in the absence of curvatures but simply by introducing an appropriate radial variation in the refractive index, it is possible to obtain converging lenses that minimize spherical aberration. The strongest evidence that spherical aberration is minimized for the GRIN lens is shown by the ray fan plot (Figure 8b) as well as by the diameter of the spot projected onto the image surface plane compared to the airy disc for the GRIN lens (Figure 9b,c).

**Figure 10:**
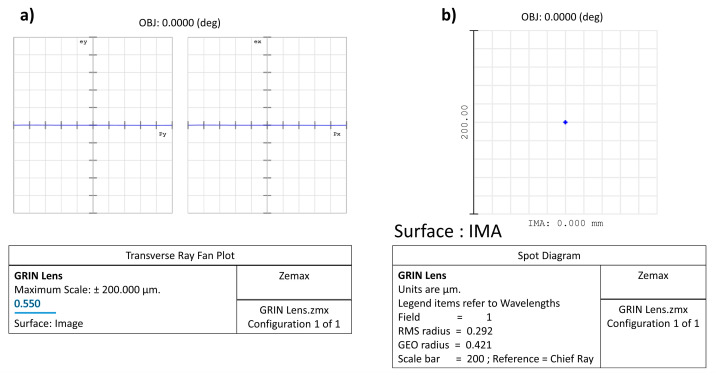
Transverse ray (**a**) and spot diagram (**b**) for the GRIN lens plotted with the same scale of the biconvex lens.

In both the transverse ray fan plot and the spot diagram graph, the scale is different for the two lenses. In the GRIN case, the scale is in fact reduced by a factor of 200 (in the absence of airy disc and 20 in the presence of airy disc). With the same scale (i.e., using the same scale of the biconvex lens also for the GRIN lens), the spot of the GRIN lens practically becomes a point, while the ray fan plot flattens out on the abscissa axis, thus showing the important reduction in spherical aberration introduced by variation alone in refractive index (Figure 10).

## 3. IOL-Based Materials Characterization and Tests of Entire IOLs

IOL material properties are consistent within the classes of IOL materials considered. This suggests that the intraoperative and postoperative behaviour of an IOL is predictable and related to its composition, thus allowing surgeons to choose IOLs more appropriate for different surgical situations and individual patient characteristics. Several diagnostic techniques are suitable to analyse IOL-based materials and also the entire IOL system. Some of these experimental techniques are also adopted to visualize laser-induced defects in hydrophilic and hydrophobic acrylic IOLs in order to obtain qualitative information on the characteristics and also analyse differences regarding IOL material and water content without damaging the lens. In the next paragraphs, some data on physico-chemical and biological tests on pristine IOL materials and the manufactured IOL are reported.

### 3.1. Physico-Chemical Characterization

During the years, the demand for improved IOLs performance in terms of flexibility, strength, and hydrophilicity has increased considering that the IOL surface influences uveal biocompatibility. The most common way to investigate IOLs hydrophobicity and biocompatibility is through contact angle measurements, whereas differential scanning calorimetry is used for evaluation of T_g_. In addition, dynamic–mechanical measures allow to achieve information on IOLs mechanical capacity and flexibility. Finally, further analyses investigating other specific properties can be accomplished by using additional methods, such as scanning electron microscopy and common spectroscopic techniques.

#### 3.1.1. Contact Angle Analyses

IOLs were characterized using contact angle (CA) measurements to assess hydrophilicity and biocompatibility. In fact, one of the most important problems affecting all IOLs materials is water capture. At equilibrium conditions, water content ranges from 0.1–0.5% for hydrophobic to 18–32% for hydrophilic polymers [2]. Non-homogeneous dispersion of water within a lens can provoke vision complications. In fact, although water molecules can freely diffuse within the lens, they can be stuck in low-density regions, acting as cavities, with subsequent formation of water micro-droplets. Such a phenomenon is the already cited “glistenings” effect [23]. Baillif et al. [85] studied IOLs produced by different manufacturers made of the following six biomaterials: collamer, hydrophilic acrylic, hydrophobic acrylic, silicone, polymethylmethacrylate (PMMA), and heparin surface-modified PMMA (HSM-PMMA). Their results, summarized in Table 3, indicate that higher hydrophobicity belongs to silicone IOLs, showing an average water contact angle of 114°, in agreement with literature values ranging from 99 to 122° [30,86,87]. The PMMA IOLs were found to have a narrower range of values, between 73.2 and 75.5°. Lenses made of hydrogel had values between 59.2 and 69.1°. The heparin-modified surface showed the lowest contact angle of 56.5°. Except for hydrophobic acrylic IOLs, showing a water contact angle (WCA) of about 88.7°, all other materials have a similar WCA between 68.4° and 79.9°. The IOLs made with hydrophilic acrylic showed the lowest WCA of about 68.4°, enabling higher water absorption on their surface [30].

It is noteworthy that the surface properties of implantable material affect the biological response of the human body [86,88,89]. For instance, one important aspect to be considered during IOLs implants is the occurrence of bacterial adhesion that can provoke inflammation and postoperative diseases. This is mainly determined by surface properties, such as wettability, in terms of hydrophobicity. The more hydrophobic a surface, the less its absorption of water (and aqueous systems). Therefore, characterization and eventual modification of IOLs surface wettability is a key parameter to reduce bacterial adhesion and thus prevent possible infections related to the used biomaterial.

Some authors hypothesized that roughness can have an effect on cells adhesion on the IOL surface, so they prepared experimental acrylic IOLs with different roughness values and examined the effect on surface cell adhesion [90]. Table 4 reports the comparison in terms of wettability and roughness properties of the 6 investigated commercial IOLs. De Giacinto et al. analysed the surface properties of these hydrophobic acrylic IOLs available in the market [91]. In detail, the authors assessed the roughness and wettability of 6 different commercial IOLs with the same dioptric power (+20.0 diopters [D]). Concerning wettability, they evaluated the WCA by means of the sessile drop method, while, for roughness, they used atomic force microscopy (AFM) with a silicone cantilever. It is noteworthy that, although made from the same material, the surface properties of IOLs change with water content and dioptric power. For instance, De Giacinto et al. considered Clareon (Model SY60WF, Alcon Laboratories, Inc.) and Vivinex iSert (Model XY1, Hoya Corp.) IOLs being both made with hydrophilic acrylic but treated in a different way. Clareon embeds more water, displaying better clarity and no glistenings, whereas Vivinex iSert is irradiated with ultraviolet-ozone for inducing on the surface material active regions with oxygenated functional groups, improving protein adsorption and cell adhesion [91,92]. As evident from Table 5, higher hydrophobicity is shown by iSert 251 (Hoya Corp.) and Vivinex iSert XY1, while Clareon SY60WF and Vivinex iSert XY1 display the smoothest surface.

Indeed, the gold standard method to determine information regarding the hydrophilic or hydrophobic nature of a substrate is measurement of the water contact angle (WCA), allowing to engage the details of the different contributions that completely characterize the surface free energy [85]. In fact, the interface properties and behaviour of a solid surface are determined by the relative amount of the main contributions to the surface energy, i.e., polar and apolar electron-donating and electron-accepting properties. Recently, for achieving information on the different interface contributions of the surface adhesion interactions [85], contact angle measurements were carried out not only with water but also with test liquids. Different models, developed at first approximation by using the Owens–Wendt calculation [93,94], have shown that the hydrophobic acrylic, silicone, and PMMA IOLs are essentially dispersive implants, whereas collamer, hydrophilic acrylic, and HSM-PMMA IOLs are polar implants [85].

#### 3.1.2. Vibrational Spectroscopic and Morphological Characterizations

**Figure 11:**
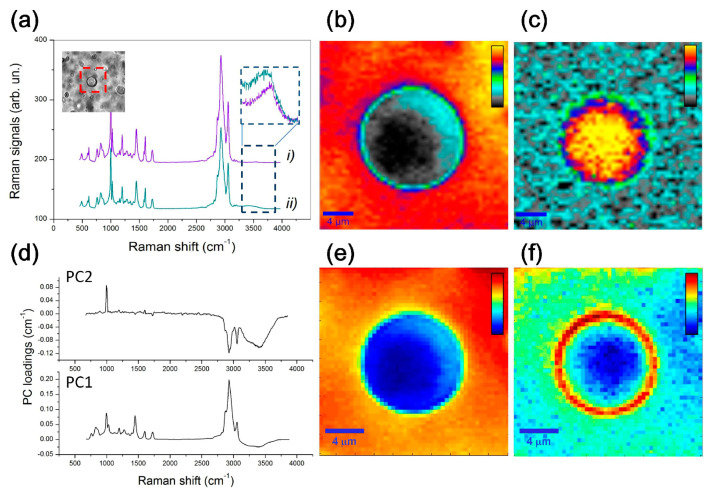
(**a**) Raman spectra obtained in a point external (***i***) and internal (***ii***) at the microvacuole. In the inset, an optical image of the microvacuole selected for Raman analysis in HFO. (**b**) Raman image of the microvacuole following the polymer band intensity (area) between 2800 and 2900 cm^−1^. (**c**) Water distribution inside the vacuole obtained by plotting the intensity of contributions in the 3200–3700 cm^−1^ range. (**d**) Loading plot for the first (bottom) and second (upper) PC, resulting from analysis of spectra acquired in a raster scan around the vacuole. (**e**,**f**) PC1 and PC2 score map from PCA of Raman spectra. Reprinted from Ref. [44] under the terms of the OSA Open Access Publishing Agreement. © 2019 Optical Society of America.

Recently, a new hydrophilic–hydrophobic membrane has been proposed as potential IOLs and contact lenses material. Membrane is based on semicrystalline s-PS (sulfonated syndiotactic polystyrene) film having sulfonated amorphous phase. The chemical and physical characteristics of the partially sulfonated (17%) s-PS membrane were investigated using several diagnostic techniques, such as SEM, Raman, FTIR, and UV–vis optical absorption. The results indicate that s-PS hydrophilicity, hydrophobicity, water content, optical clarity, mechanical, and adhesive and permeability properties make this material potentially useful for intraocular lens (IOLs) and contact lens applications [95]. Rusciano et al. [44] combine optical microscopy with micro-Raman spectroscopy to analyse limited portions of the IOL around a single microvacuole. Two different IOL materials were analysed: the first is a common hydrophobic acrylate copolymer, while the second one differs regarding presence of a percentage of hydrophilic polymer component. In Figure 11 are reported Raman spectra and Raman mapping on a selected microvacuole following the hydrophobic foldable ocular (HFO) lens material and then the water signals and PCA analysis results. 

From these data, it emerged that vacuoles are not water-filled pockets (the estimated water concentration inside a microvacuole is 23%) and mainly that, in presence of a well-distributed hydrophilic component, water is prone to be spread over the whole IOL polymeric matrix, therefore avoiding trapping by phenyl-rings-rich environments. Raman features have also been followed to identify the changes induced by a photochemical process, wherein hydrophilic polar functional groups are generated by photo-induced hydrolysis of polymeric materials in areas that are exposed to a femtosecond laser, thus providing the chemical basis for a hydrophilicity-based refractive index change, facilitating creation of a refractive index shaping lens (RIS-lens) [82]. 

The distribution of nano-microstructure of sediments on the IOL surface was generally determined using optical microscopy and AFM, while the surface composition and chemical bonding configurations of the IOL materials were determined carrying out X-ray photoelectron spectroscopy (XPS) [96]. Nanopatterning of IOLs surface can suppress upregulation of cytoskeleton proteins (actin and actinin) within the cells in contact with the IOL surface and, hence, prevent secondary cataracts causing blurry or opaque vision. One of the most nanopatterned synthetic hydrogels is based on poly (2-hydroxyethyl methacrylate, PHEMA). PHEMA is a transparent, biocompatible, nontoxic, non-degradable, non-adhesive, hydrophilic hydrogel material with high and tuneable mechanical strength, largely applied in ophthalmology. This polymer has been synthesized by Tomáš Krajňák et al. [97] from the precursor monomer 2-hydroxyethyl methacrylate (HEMA) via thermally or radiatively (gamma, UV, blue-light) initiated free radical polymerization. XPS and FTIR analyses were employed to identify chemical bonds within the unmodified and patterned PHEMA surface, with results unchanged after the patterning. The investigated imprinted nanostructured hydrogels mimic the natural cell environment. Thus, they are interesting materials to manufacture future innovative IOLs.

Finally, we remark that, in recent decades, the interest in IOLs with ultraviolet (UV) and blue-light filters has increased [98]. The natural crystalline lens absorbs light of wavelengths between 300 and 400 nm, and several researchers theorize that the crystalline should be replaced with an IOL that includes a UV-light-blocking chromophore [98]. In 1978, Mainster investigated the sensitivity of the pseudophakic eye to UV light and possible related retinal damage [99]. At the same time, blue-blocking IOLs were introduced to prevent the risk of age-related macular degeneration (AMD) [100]. In order to block the blue wavelength light, yellow-tinted IOLs were launched on the market in the 1990s [98,100]. Whether introduction of the blue-light filter is a benefit or harm for the patient is still debated today. Several studies have been published with controversial results. Artigas et al. [101] compared the spectral transmittance of ten different IOLs in order to investigate the performance of their UV or blue-light filters with sunlight or artificial light. The action of the filters is not the same: (1) filters that favour better photoreception of visible light (380–780 nm) are those that transmit all the radiation (100%); (2) filters that provide greater photoprotection against UV radiation, even blue light, are yellow and orange. The blue-blocking IOLs are advantageous in terms of visual acuity and contrast sensitivity, but some aspects, such as the benefits of blocking the transmission of blue light to the macula, are not very well known [100].

#### 3.1.3. Mechanical and Thermomechanical Tests

During the design and developing phases, it is important to consider IOLs stability, strongly dependent on the mechanical properties of the lens, affecting the outcome of cataract surgery. The aim is to avoid residual refractive errors and other complications. For this reason, the manufacturer of IOLs must test the mechanical performances before obtaining the conformity certification required for placing on the market. A reference used for these tests is the international standard ISO 11979-3 published in 2012 [102]. The recommended tests involve measurement of compression force, measurement of axial displacement in compression, of optic decentration and optic tilt, measurements of angle of contact and loop pull strength, as well as testing of compression force decay and dynamic fatigue durability.

In the literature, we found some papers regarding the strain–stress characteristics of both hydrophobic and hydrophilic materials estimated experimentally and simulated using finite-element modelling [103]. Lane et al. [104] evaluated the axial displacement, optic decentration, and optic tilt of five IOL models, investigating the consistency of the possible refractive outcomes. Furthermore, after cataract surgery, it is fundamental to evaluate IOL tilt and decentration. Gu et al. [105] observed that the tilt and decentration of postoperative IOLs are greater in patients with more tilted and decentred crystalline lenses, as well as those with shorter or longer axial length. 

The effect of intraocular lens tilt and decentration on visual quality partly depends on the thermomechanical properties of the materials constituting implantable IOLs. Therefore, investigation of these properties is a fundamental task because it may determine degradation under physiological conditions of the considered material. The mechanical properties of polymers strongly change with temperature. In particular, they appear soft and are able to flow at temperatures higher than the so-called glass transition temperature (T_g_) [23,40]. Instead, they appear hard for T < T_g_. Indeed, materials with a lower T_g_ are more deformable and easily foldable at the body temperature at which the IOL is implanted [106]. For instance, lenses made by PMMA display glass-like behaviour, having a T_g_ of about 110 °C, whereas those made by silicone display rubber-like features and rapidly unfold within the eye because they have a T_g_ in the range −119.6 °C < T_g_ <−91.7 °C. Acrylic IOLs have Tg values in between those made by PMMA and silicone, displaying T_g_ values from 14 to 15.5 °C [30]. For glass transition temperatures higher than body temperature, the polymer (or copolymer) must have a low water content to be flexible because, in these cases, water acts as a plasticizer [107]. T_g_ is usually measured by means of differential scanning calorimetry (DSC) measurements, which is an experimental technique able to determine, by looking at differences in heat flow with respect to a control sample, eventual phase transitions occurring in an investigated sample by varying the temperature. Furthermore, thermogravimetric analysis (TGA) enables evaluation of variations in weight due to variations in temperature, allowing to achieve polymer degradation temperature, absorbed moisture content of materials, or solvent residues [108].

**Figure 12:**
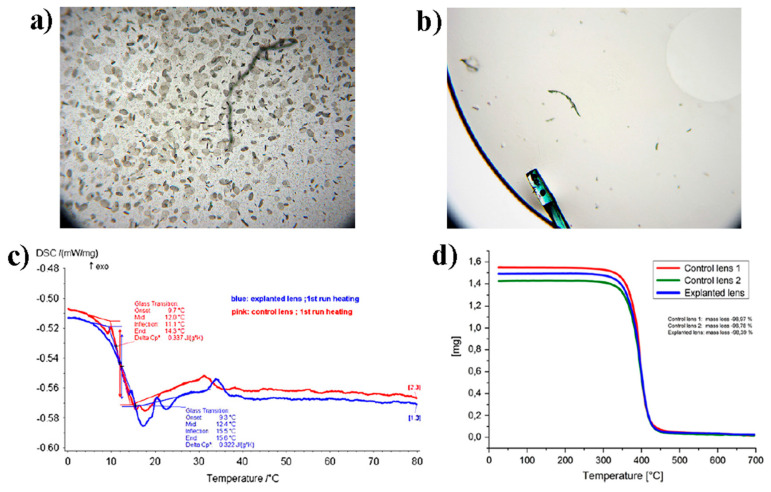
Comparison between two IOLs made of hydrophobic acrylic. (**a**) Light photomicrographs at 40x magnification showing intra-optic glistening for an IOL explanted from a patient 26 days after implantation. (**b**) Same as (**a**) but for a control IOL kept in a balanced salt solution at 37 °C for 2 weeks showing only crystal-like deposits. (**c**) DSC curves for explanted and control IOLs for determination of Tg (about 12 °C in both cases), showing non-perfect overlap. (**d**) TGA curves showing very high stability for all IOLs; in this case, two control IOLs have been considered. Figure adapted with permission from Ref. [108]. L. Werner et al. Unusual Pattern of Glistening Formation on a 3-Piece Hydrophobic Acrylic Intraocular Lens. *J*. *Cataract. Refract. Surg.* **2008**, *34*, 1604–1609. © Wolters Kluwer Health, Inc., Philadelphia, PA, USA.

Figure 12 reports a comparison between two hydrophobic acrylic IOLs, one explanted from a patient 26 days after implantation due to glistenings and one as a control IOL kept in a balanced salt solution at 37 °C for 2 weeks. Light photomicrographs at 40× magnification show intra-optic glistening for the explanted IOL (Figure 12a), whereas only crystal-like deposits on the control IOL (Figure 12b). DSC and TGA analyses instead show only slight or no differences in the corresponding thermograms. Although the DSC curves do not overlap perfectly, the evaluated T_g_ is essentially the same, about 12 °C (Figure 12c). TGA analysis shows that the IOLs constituting material is stable up to about 250 °C, indicating absence of any unreacted monomers or solvents (Figure 12d). The high glistenings level shown by the explanted IOLs is probably enhanced by surgical stress and temperature fluctuations that indeed play a very important role for choosing and treatment of IOLs constituting material [108].

The glistenings issue, in terms of water accumulation in lower-density ‘‘pockets’’ that expand over time, is strictly related to polymer water content that can be affected by temperature variation [22]. Therefore, complete control of mechanical and thermomechanical properties of the material constituting the IOL is fundamental. Table 5 reports some of the most relevant physical properties for conventional IOL materials, including new hydrophobic acrylics constituted by a copolymer of three different monomers PEG-PEA/HEMA/styrene (PHS) and a crosslinker. The PEG chains provide high flexibility, the styrene moiety allows enhancing the refractive index, HEMA moiety allows dispersion in water of the material, and the crosslinker (ethylene glycol dimethacrylate) reinforces the structure. The tensile stress value corresponds to the highest stress that the material can tolerate while being pulled without cracking [23].

### 3.2. Biocompatibility

The safety of an intraocular lens is strongly dependent on the biocompatibility of the employed material. The risk of toxicity, genotoxicity, irritation, and inflammation must be evaluated both in vitro and in vivo in compliance with ISO 11979-5:2020 [109]. The tests in vitro are required for possible adverse events due to the chemical compounds that could release small particles after implantation [110]. The final product is tested both for eventual extractables and leachables: the former ones are compounds that can be extracted from the material if it is in a specific environment (solvent) at pre-established temperature and time of exposure (usually worse conditions than end use); the latter ones involve any possible compound that could migrate inside the medical device during production or storage [110]. Any insoluble inorganic residue due to IOL manufacturing must be identified and evaluated [111]. Generally, the longevity of an IOL is evaluated in accelerated conditions thanks to a test for hydrolytic stability and a photostability test. By using high-temperature and high-UV power intensity exposure, aging of 20 years is simulated and the long-term stability can be studied in terms of the altered properties of the “aged” sample [111,112]. Moreover, an IOL must be resilient to neodymium:yttrium aluminium garnet (Nd:YAG) laser and a test to its exposure must be carried out. This is because Nd:YAG laser capsulotomy is a widely used technique as treatment for posterior capsular opacification (PCO). The manufacturer must ensure that no leakage from IOL or physicochemical effect occur after laser exposure [110]. 

It is equally important to define degree of biocompatibility, usually unpredictable. Cytotoxicity is often evaluated by considering the effects on L-929 mouse fibroblast cell lines due to exposure to the material under consideration or its possible extractables/leachables [110]. Werner et al. studied the cytotoxicity of Teflon-coated PMMA in terms of qualitative morphological evaluation and neutral red extraction assay and did not find any toxic effect [113]. Kao et al. proposed an alternative method and tested cytotoxicity of silicone discs used in ophthalmic field by exposing human lens epithelial cells (HLEC) to toxic compounds [114]. Tortolano et al. [115] investigated cytotoxicity of four commercial IOLs and quantified the amount of fibronectin adsorption on the IOL surface. The IOLs under investigation were made of hydrophobic acrylate materials. They did not find any toxic effect, even with simulated aging of 2 years [115]. In Table 6, the advantages and disadvantages in terms of biocompatibility of IOLs materials are reported.

Generally, biological tests for sensitization and irritation are carried out with animals, specifically with guinea pigs for the former and albino rabbits for the latter [110]. Rabbits are also used to study the tissue response to IOL materials after implantation [110]. The inflammatory response is evaluated both short-term and long-term [110]. Finally, genotoxicity tests are required to evaluate eventual gene mutations and/or chromosomal damage due to IOL implantation and are usually carried out in vitro [110]. Generally, bacterial reverse mutation test (AMES) or mouse lymphoma assay (MLA) are used to evaluate gene mutations. Similarly, for in vitro chromosomal damage test, MLA or chromosomal aberration (CA) assay can be used [110]. If a genotoxic response is obtained from any of these in vitro tests, a genotoxicity test in vivo is required. Commonly, in vivo mouse micronucleus assay or in vivo chromosomal aberration assay are carried out to investigate possible genotoxic effects of IOL materials [110]. 

## 4. Conclusions

In this contribution, the state of the art of polymeric systems and their limitations for future IOL improvements are reported. The main goal of modern cataract surgery is to be able to employ the smallest incision possible, so the IOLs had to be flexible. This goal was achieved by improvements in the IOL design and materials that made them foldable. Improved physico-chemical properties and functions have been obtained by adding extra polymers or surface modification. Nowadays, high-quality hybrid polymers, such as PEG-PEA/HEMA/Styrene or 2-HEMA and EOEMA copolymers, are studied to improve biocompatibility, also providing better visual quality and adjustable ability, reducing surgical incision, as well as dealing with complications such as posterior capsular opacification (PCO). Recently, creation of ophthalmic optics by combining several materials with different refractive indexes (GRIN) useful to create patient-specific eye power is of primary importance. The main advantage of GRIN-based IOLs is that they would be much more anatomically similar to the crystalline lens than the current technology with single material. Despite the great results obtained, new challenges are underway, exploiting developments of new techniques and thanks to the availability of advanced materials with improved properties. However, the need for cheaper and more biocompatible materials never ends. Actual research leads to developing materials with unique features and specific functionality. However, further efforts should be made for understanding the structure–property correlation in terms of physico-mechanical properties (such as glass transition temperature, wettability, flexibility, oxygen permeability), chemical properties (such as stability, inertness), and biochemical properties (such as biocompatibility), which will enable scientists to ascertain other innovative materials with desired properties and behaviour.

## Figures and Tables

**Table 1:** IOL-based materials and some of their properties.

	Silicone	PMMA	Hydrophobic Acrylic	Hydrophilic Acrylic
Rigidity	flexible	rigid	flexible	flexible
Affinity to water	hydrophobic	hydrophobic	hydrophobic	hydrophilic
Refractive index	1.41–1.46	1.49	up to 1.55	1.43
Abbe number	42	58	37–55	58

**Table 3:** Values of the average water contact angles (in ° +/− the standard deviation, SD) on the tested IOLs materials.

Material	WCA (° +/− SD)
Collamer	77.7 +/− 0.79
Hydrophilic acrylic	68.4 +/− 1.11
Hydrophobic acrylic	88.7 +/− 1.24
Silicone	114.1 +/− 2.54
PMMA	77.1 +/− 2.34
Heparinized PMMA	79.9 +/− 2.21

**Table 4:** Drop profiles for evaluation of the WCA for the 6 commercial considered IOLs and corresponding root mean square roughness measured by AFM. Adapted with permission from Ref. [91] C. de Giacinto et al. Surface Properties of Commercially Available Hydrophobic Acrylic Intraocular Lenses: Comparative Study, *J. Cataract Refract. Surg.* **2019**, *45*, 1330–1334. © Wolters Kluwer Health, Inc., Philadelphia, PA, USA.

Model	Drop Profiling	WCA (°)	RMS Roughness (nm)
iSert 251	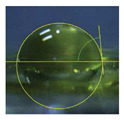	84.24 +/− 2.15	1.78 +/− 0.40
CT Lucia 601P	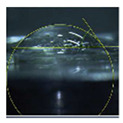	48.76 +/− 4.91	2.05 +/− 0.36
enVista MX60	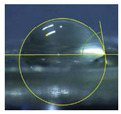	75.22 +/− 3.45	2.13 +/− 0.26
Clareon SY60WF	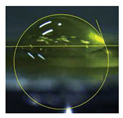	72.84 +/− 1.80	1.01 +/− 0.41
Vivinex iSert XY1	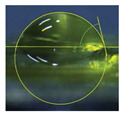	78.94 +/− 6.66	0.63 +/− 0.57
Tecnis PCB00	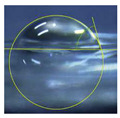	74.45 +/− 5.11	2.27 +/− 2.54

**Table 5:** Relevant physical properties for conventional IOL materials. Data from New Hydrophobic IOL Materials and Understanding the Science of Glistenings, Tetz, M.; Jorgensen, M.R. Curr Eye Res 2015, 40, 969–981 [23], reprinted with permission of the publisher (Taylor & Francis Ltd., http://www.tandfonline.com, accessed on 2 March 2023).

Material	Water Content (%)	WCA (°)	Tensile Stress (MPa)	*n*	Tg (°C)
PMMA	0.4–0.8	65–71	47–70	1.49	105–113
Silicone	0.38	97–120	5.9–8.2	1.43	−120–−90
Hydrophilic acrylics	18–38	20–70	0.4.06	1.40–1.43	10–20
Hydrophobic acrylics	0.1–0.5	72–88	No data	1.47–1.56	5–16
PHS	4–5	69–79	4–6	1.54	27–29

**Table 6:** Comparison of biocompatibility of IOL materials. Reprinted with permission from Ref. [116].

Materials	Advantage	Disadvantage
Hydrophilic acrylic	Higher tissue compatibility due to high water content; Low aqueous flare; Low rate of inflammatory cell accumulation on the lens surface.	Insufficient posterior sharp-edged design due to the high water content; High rate of posterior capsule opacification; High rate of anterior capsule opacification; Greater lens epithelial cell on growth on the lens surface.
Hydrophobic acrylic	Material compatible with a posterior sharp-edged design; Low rate of posterior capsule opacification; Low rate of anterior capsule opacification; Low rate of lens epithelial cell on growth on the lens surface.	High aqueous flare * Inflammatory cell accumulation on the lens surface *. * Not at a clinically significant level
PMMA	Good tissue compatibility; Low aqueous flare; Low rate of inflammatory cell accumulation on the lens surface.	Foldable High rate of posterior capsule opacification
Silicone	Low rate of inflammatory cell accumulation on the lens surface; Low rate of posterior capsule opacification.	Increased fibrotic reaction due to lens epithelial cell stimulation; Lens surface opacification due to contact with intravitreal air; Difficulty visualizing the retina due to interface formed with silicone oil used in vitreoretinal surgery.

## Data Availability

The new simulation data reported in this review are available upon reasonable request to the corresponding author.

## References

[1] National Eye Institute Cataracts. https://www.nei.nih.gov/learn-about-eye-health/eye-conditions-and-diseases/cataracts.

[2] Luo C., Wang H., Chen X., Xu J., Yin H., Yao K. (2022). Recent Advances of Intraocular Lens Materials and Surface Modification in Cataract Surgery. Front. Bioeng. Biotechnol..

[3] de Groot J.H., van Beijma F.J., Haitjema H.J., Dillingham K.A., Hodd K.A., Koopmans S.A., Norrby S. (2001). Injectable Intraocular Lens Materials Based upon Hydrogels. Biomacromolecules.

[4] Assia E.I., Blumenthal M., Apple D.J. (1999). Effect of Expandable Full-Size Intraocular Lenses on Lens Centration and Capsule Opacification in Rabbits. J. Cataract Refract. Surg..

[5] Song L., Hu W., Wang G., Zhang H., Niu G., Cao H., Yang H., Zhu S. (2011). Synthesis and Characterization of Shape Memory (Meth)Acrylate Co-Polymers and Their Cytocompatibility In Vitro. J. Biomater. Sci. Polym. Ed..

[6] Ford J., Werner L., Mamalis N. (2014). Adjustable Intraocular Lens Power Technology. J. Cataract Refract. Surg..

[7] Werner L., Chang W., Haymore J., Haugen B., Romaniv N., Sandstedt C., Chang S., Mamalis N. (2010). Retinal Safety of the Irradiation Delivered to Light-Adjustable Intraocular Lenses Evaluated in a Rabbit Model. J. Cataract Refract. Surg..

[8] Bille J.F., Engelhardt J., Volpp H.-R., Laghouissa A., Motzkus M., Jiang Z., Sahler R. (2017). Chemical Basis for Alteration of an Intraocular Lens Using a Femtosecond Laser. Biomed. Opt. Express.

[9] Zhong X., Long E., Chen W., Xiang W., Liu Z., Chen H., Chen J., Lin Z., Lin H., Chen W. (2016). Comparisons of the In-the-Bag Stabilities of Single-Piece and Three-Piece Intraocular Lenses for Age-Related Cataract Patients: A Randomized Controlled Trial. BMC Ophthalmol..

[10] Zvorničanin J., Zvorničanin E. (2018). Premium Intraocular Lenses: The Past, Present and Future. J. Curr. Ophthalmol..

[11] Roach L., Pepose J.S., Santhiago M.R., Waring G.O. Centration of IOLs: Challenges, Variables, and Advice for Optimal Outcomes. https://www.aao.org/eyenet/article/centration-of-iols-challenges-variables-advice-opt.

[12] Wang L., Houser K., Koch D.D., Yanoff M., Duker J.S. (2019). Intraocular Lens Power Calculations. Ophthalmology.

[13] Werner L. (2021). Intraocular Lenses. Ophthalmology.

[14] Megiddo-Barnir E., Alió J.L. (2023). Latest Development in Extended Depth-of-Focus Intraocular Lenses: An Update. Asia-Pac. J. Ophthalmol..

[15] Sánchez-González J.-M., Sánchez-González M.C., De-Hita-Cantalejo C., Ballesteros-Sánchez A. (2022). Small Aperture IC-8 Extended-Depth-of-Focus Intraocular Lens in Cataract Surgery: A Systematic Review. J. Clin. Med..

[16] Pérez-Vives C. (2018). Biomaterial Influence on Intraocular Lens Performance: An Overview. J. Ophthalmol..

[17] Thomes B.E., Callaghan T.A. (2013). Evaluation of in Vitro Glistening Formation in Hydrophobic Acrylic Intraocular Lenses. Clin. Ophthalmol..

[18] Dhital A., Spalton D.J., Goyal S., Werner L. (2012). Calcification in Hydrophilic Intraocular Lenses Associated with Injection of Intraocular Gas. Am. J. Ophthalmol..

[19] Oshika T. (2001). Influence of Glistenings on the Optical Quality of Acrylic Foldable Intraocular Lens. Br. J. Ophthalmol..

[20] Zhao H., Mainster M.A. (2007). The Effect of Chromatic Dispersion on Pseudophakic Optical Performance. Br. J. Ophthalmol..

[21] Pinchuk L. (2022). The Use of Polyisobutylene-Based Polymers in Ophthalmology. Bioact. Mater..

[22] Werner L. (2010). Glistenings and Surface Light Scattering in Intraocular Lenses. J. Cataract Refract. Surg..

[23] Tetz M., Jorgensen M.R. (2015). New Hydrophobic IOL Materials and Understanding the Science of Glistenings. Curr. Eye Res..

[24] Mao Y., Liu H., Long Gu F., Wu M.-X., Wang Y. (2022). The Molecular Design of Performance-Enhanced Intraocular Lens Composites. Biomater. Sci..

[25] Nagata T., Kubota S., Watanabe I., Aoshima S. (1999). Chromatic Aberration in Pseudophakic Eyes. Nippon Ganka Gakkai Zasshi.

[26] Smith G., Atchison D.A. (1996). The Eye and Visual Optical Instruments.

[27] Jellali R., Alexandre M., Jérôme C. (2017). Photosensitive Polydimethylsiloxane Networks for Adjustable-Patterned Films. Polym. Chem..

[28] Teshigawara T., Meguro A., Mizuki N. (2021). Relationship between Postoperative Intraocular Lens Shift and Postoperative Refraction Change in Cataract Surgery Using Three Different Types of Intraocular Lenses. Ophthalmol. Ther..

[29] Shimizu K., Kobayashi K., Takayama S., Zhaobin G. (2008). Preloaded Injector for Intraocular Lens Implantation without the Use of Ophthalmic Viscosurgical Devices. J. Cataract Refract. Surg..

[30] Tehrani M., Dick H.B., Wolters B., Pakula T., Wolf E. (2004). Material Properties of Various Intraocular Lenses in an Experimental Study. Ophthalmologica.

[31] Li N., Chen X., Zhang J., Zhou Y., Yao X., Du L., Wei M., Liu Y. (2008). Effect of AcrySof versus Silicone or Polymethyl Methacrylate Intraocular Lens on Posterior Capsule Opacification. Ophthalmology.

[32] Bozukova D., Pagnoulle C., Jérôme R., Jérôme C. (2010). Polymers in Modern Ophthalmic Implants—Historical Background and Recent Advances. Mater. Sci. Eng. R Rep..

[33] Wei Y., Chen Y., Liu P., Gao Q., Sun Y., Huang C. (2011). Surface Modification of Hydrophobic PMMA Intraocular Lens by the Immobilization of Hydroxyethyl Methacrylate for Improving Application in Ophthalmology. Plasma Chem. Plasma Process..

[34] Kohnen T. (1996). The Variety of Foldable Intraocular Lens Materials. J. Cataract Refract. Surg..

[35] Bellucci R., Güell J.L. (2013). An Introduction to Intraocular Lenses: Material, Optics, Haptics, Design and Aberration. Cataract. ESASO Course Series.

[36] Lee H., Tae G., Kim Y.H. (2008). A Study on the Copolymerization Kinetics of Phenylethyl Acrylate and Phenylethyl Methacrylate. Macromol. Res..

[37] Parra Ruiz F.J., Vazquez Lasa B., San Roman Del Barrio J. (2010). Hydrophilic Acrylic Systems with a High Refractive Index for Producing Intraocular Lenses.

[38] Murthy K.S., Ravi N. (2001). Hydrogels as Potential Probes for Investigating the Mechanism of Lenticular Presbyopia. Curr. Eye Res..

[39] Hoffman A.S. (2001). Hydrogels for Biomedical Applications. Ann. N. Y. Acad. Sci..

[40] Tripti D., Haldar R.S., Geetha S., Niyogi U.K., Khandal R.K. (2009). Materials for Intraocular Lenses (IOLs): Review of Developments to Achieve Biocompatibility. e-Polymers.

[41] Fernandes P., González-Méijome J.M., Madrid-Costa D., Ferrer-Blasco T., Jorge J., Montés-Micó R. (2011). Implantable Collamer Posterior Chamber Intraocular Lenses: A Review of Potential Complications. J. Refract. Surg..

[42] Seward H.C. (1997). Folding Intraocular Lenses: Materials and Methods. Br. J. Ophthalmol..

[43] Bellone A. Mini Well Ready Iol. https://albertobellone.it/en/mini-well-ready-iol-sifi-medtech/.

[44] Rusciano G., Capaccio A., Pesce G., Sasso A. (2019). Experimental Study of the Mechanisms Leading to the Formation of Glistenings in Intraocular Lenses by Raman Spectroscopy. Biomed. Opt. Express.

[45] Werner L., Legeais J.-M., Nagel M.-D., Renard G. (1999). Evaluation of Teflon-Coated Intraocular Lenses in an Organ Culture Method. J. Biomed. Mater. Res..

[46] Ratner B.D., Mateo N.B. (1987). Polymeric Intraocular Lens Material Having Improved Surface Properties 1987.

[47] Lee H.I., Kim M.K., Ko J.H., Lee H.J., Wee W.R., Lee J.H. (2007). The Efficacy of an Acrylic Intraocular Lens Surface Modified with Polyethylene Glycol in Posterior Capsular Opacification. J. Korean Med. Sci..

[48] Hoffman A.S., Patel A.S., Llanos G. (1997). Polyethylene Oxide Coated Intraocular Lens.

[49] Bozukova D., Pagnoulle C., De Pauw-Gillet M.-C., Desbief S., Lazzaroni R., Ruth N., Jérôme R., Jérôme C. (2007). Improved Performances of Intraocular Lenses by Poly(ethylene glycol) Chemical Coatings. Biomacromolecules.

[50] Bozukova D., Pagnoulle C., De Pauw-Gillet M.-C., Ruth N., Jérôme R., Jérôme C. (2008). Imparting Antifouling Properties of Poly(2-hydroxyethyl methacrylate) Hydrogels by Grafting Poly(oligoethylene glycol methyl ether acrylate). Langmuir.

[51] Tognetto D., Toto L., Minutola D., Ballone E., di Nicola M., Di Mascio R., Ravalico G. (2003). Hydrophobic Acrylic versus Heparin Surface-Modified Polymethylmethacrylate Intraocular Lens: A Biocompatibility Study. Graefe’s Arch. Clin. Exp. Ophthalmol..

[52] Larsson R., Selen G., Bjorklund H., Fagerholm P. (1989). Intraocular PMMA Lenses Modified with Surface-Immobilized Heparin: Evaluation of Biocompatibility In Vitro and In Vivo. Biomaterials.

[53] González-Chomón C., Concheiro A., Alvarez-Lorenzo C. (2011). Drug-Eluting Intraocular Lenses. Materials.

[54] Zhang L., Schickhardt S., Fang H., Auerbach F., Cagampang P., Merz P.R., Auffarth G.U. (2022). Comparison of a New IOL Injector System against 3 Standard IOL Injector Systems with Different Incision Sizes: Miyake-Apple View Experimental Laboratory Study. J. Cataract Refract. Surg..

[55] Cabeza-Gil I., Ríos-Ruiz I., Calvo B. (2021). Experimental Evaluation of the Injection Force Exerted in Intraocular Lens Delivery with Syringe-Type Injectors. J. Mech. Behav. Biomed. Mater.

[56] Lendlein A., Kelch S. (2002). Shape-Memory Polymers. Angew. Chem. Int. Ed..

[57] Oh W.T., Lee J.B., Choi W., Bae H.W., Kim C.S., Kim C.Y., Sung H.-J. (2020). Shape Memory Tube Plug for Fine-Control of Intraocular Pressure by Glaucoma Devices. ACS Biomater Sci. Eng..

[58] El Feninat F., Laroche G., Fiset M., Mantovani D. (2002). Shape Memory Materials for Biomedical Applications. Adv. Eng. Mater..

[59] Kimura W., Kimura T., Sawada T., Kikuchi T., Toda H., Yamada Y., Nagai H. (1991). Comparison of Shape Recovery Ratios in Various IOL Haptics. Nippon Ganka Gakkai Zasshi.

[60] Scholl J., Smiley T., Smith D.J., Burns D.H., Cheskin B. (2013). Accommodating Intraocular Lens System Having Circumferential Haptic Support and Method.

[61] Kahook M.Y., Mandava N., Shandas R., Rech B., Lowery M.D., Urbaniak D. (2014). Shape Memory Polymer Intraocular Lenses.

[62] Patadiya J., Gawande A., Joshi G., Kandasubramanian B. (2021). Additive Manufacturing of Shape Memory Polymer Composites for Futuristic Technology. Ind. Eng. Chem. Res..

[63] Lichtinger A. (2012). The Light Adjustable Lens—A Review. Eur. Ophthalmic Rev..

[64] Light-Adjustable IOLs & Cataracts with Unusual Corneas. https://ophthalmologybreakingnews.com/ophthalmologynews-light-adjustable-iols-cataractsunusualcorneas.

[65] RxSight Inc. Summary of Safety and Effectiveness Data (SSED). https://www.accessdata.fda.gov/cdrh_docs/pdf16/P160055B.pdf.

[66] Chang D.F. (2019). Disruptive Innovation and Refractive IOLs: How the Game Will Change with Adjustable IOLs. Asia-Pac. J. Ophthalmol..

[67] Schojai M., Schultz T., Schulze K., Hengerer F.H., Dick H.B. (2020). Long-Term follow-up and Clinical Evaluation of the Light-Adjustable Intraocular Lens Implanted after Cataract Removal: 7-Year Results. J. Cataract Refract. Surg..

[68] Fernandez E.J., Artal P. (2017). Achromatic Doublet Intraocular Lens for Full Aberration Correction. Biomed. Opt. Express.

[69] Malyugin B., Morozova T., Cherednik V. (2014). Gradient Refractive Index Optics IOL: Theoretical Background and Clinical Results. MEAJO Middle East Afr. J. Ophthalmol..

[70] Pierscionek B.K., Regini J.W. (2012). The Gradient Index Lens of the Eye: An Opto-Biological Synchrony. Prog. Retin. Eye Res..

[71] Ruan X., Liu Z., Luo L., Liu Y. (2020). The Structure of the Lens and Its Associations with the Visual Quality. BMJ Open Ophthalmol..

[72] Liu Y.-J., Wang Z.-Q., Song L.-P., Mu G.-G. (2005). An Anatomically Accurate Eye Model with a Shell-Structure Lens. Optik.

[73] Navarro R. (2009). The Optical Design of the Human Eye: A Critical Review. J. Optom..

[74] Chang Y.-C., Mesquita G.M., Williams S., Gregori G., Cabot F., Ho A., Ruggeri M., Yoo S.H., Parel J.-M., Manns F. (2019). In Vivo Measurement of the Human Crystalline Lens Equivalent Refractive Index Using Extended-Depth OCT. Biomed. Opt. Express.

[75] Feng Y., Lin Y., Xiong S., Xu X. Electromagnetic Wave Lenses and Reflectors Designed with Transformation Electromagnetics. Proceedings of the 2014 XXXIth URSI General Assembly and Scientific Symposium (URSI GASS).

[76] Ohmi S., Sakai H., Asahara Y., Nakayama S., Yoneda Y., Izumitani T. (1988). Gradient-Index Rod Lens Made by a Double Ion-Exchange Process. Appl. Opt..

[77] Ellis J.D., Brooks D.R., Wozniak K.T., Gandara-Montano G.A., Fox E.G., Tinkham K.J., Butler S.C., Zheleznyak L.A., Buckley M.R., Funkenbusch P.D. Manufacturing of Gradient Index Lenses for Ophthalmic Applications. Proceedings of the Optical Design and Fabrication 2017 (Freeform, IODC, OFT).

[78] Pickering M.A., Taylor R.L., Moore D.T. (1986). Gradient Infrared Optical Material Prepared by a Chemical Vapor Deposition Process. Appl. Opt..

[79] Sinai P. (1971). Correction of Optical Aberrations by Neutron Irradiation. Appl. Opt..

[80] Ji S., Yin K., Mackey M., Brister A., Ponting M., Baer E. (2013). Polymeric Nanolayered Gradient Refractive Index Lenses: Technology Review and Introduction of Spherical Gradient Refractive Index Ball Lenses. Opt. Eng..

[81] Sola D., Cases R. (2020). High-Repetition-Rate Femtosecond Laser Processing of Acrylic Intra-Ocular Lenses. Polymers.

[82] Sahler R., Bille J.F., Bille J. (2019). Refractive Index Shaping: In Vivo Optimization of an Implanted Intraocular Lens (IOL). High Resolution Imaging in Microscopy and Ophthalmology.

[83] Sahler R., Bille J.F., Enright S., Chhoeung S., Chan K. (2016). Creation of a Refractive Lens within an Existing Intraocular Lens Using a Femtosecond Laser. J. Cataract Refract. Surg..

[84] Almeida M.S.d., Carvalho L.A. (2007). Different Schematic Eyes and Their Accuracy to the in Vivo Eye: A Quantitative Comparison Study. Braz. J. Phys..

[85] Baillif S., Baziard-Mouysset G., Roques C., Baziard Y., Kodjikian L. (2013). Calculation of Intraocular Lens Surface Free Energy and Its Components from Contact Angle Measurements. Ophthalmic Res..

[86] Cunanan C.M., Ghazizadeh M., Buchen S.Y., Knight P.M. (1998). Contact-Angle Analysis of Intraocular Lenses. J. Cataract Refract. Surg..

[87] Dick H.B., Frohn A., Augustin A.J., Wolters B., Pakula T., Pfeiffer N. (2001). Physicochemical Surface Properties of Various Intraocular Lenses. Ophthalmic Res..

[88] Lydon M.J., Minett T.W., Tighe B.J. (1985). Cellular Interactions with Synthetic Polymer Surfaces in Culture. Biomaterials.

[89] Andrade J.D., Hlady V. (1986). Protein Adsorption and Materials Biocompatibility: A Tutorial Review and Suggested Hypotheses.

[90] Tanaka T., Shigeta M., Yamakawa N., Usui M. (2005). Cell Adhesion to Acrylic Intraocular Lens Associated with Lens Surface Properties. J. Cataract Refract. Surg..

[91] De Giacinto C., Porrelli D., Turco G., Pastore M.R., D’Aloisio R., Tognetto D. (2019). Surface Properties of Commercially Available Hydrophobic Acrylic Intraocular Lenses: Comparative Study. J. Cataract Refract. Surg..

[92] Matsushima H., Iwamoto H., Mukai K., Obara Y. (2006). Active Oxygen Processing for Acrylic Intraocular Lenses to Prevent Posterior Capsule Opacification. J. Cataract Refract. Surg..

[93] Owens D.K., Wendt R.C. (1969). Estimation of the Surface Free Energy of Polymers. J. Appl. Polym. Sci..

[94] Altay B.N., Ma R., Fleming P.D., Joyce M.J., Anand A., Chen T., Keskin B., Maddipatla D., Turkani V.S., Kotkar P.R. (2020). Surface Free Energy Estimation: A New Methodology for Solid Surfaces. Adv. Mater. Interfaces.

[95] Zuppolini S., Borriello A., Pellegrino M., Venditto V., Ambrosio L., Nicolais L. (2017). Potential Contact and Intraocular Lenses Based on Hydrophilic/Hydrophobic Sulfonated Syndiotactic Polystyrene Membranes. J. King Saud Univ. Sci..

[96] Tarnawska D., Balin K., Jastrzębska M., Talik A., Wrzalik R. (2020). Physicochemical Analysis of Sediments Formed on the Surface of Hydrophilic Intraocular Lens after Descemet’s Stripping Endothelial Keratoplasty. Materials.

[97] Krajňák T., Černá E., Šuráňová M., Šamořil T., Zicha D., Vojtová L., Čechal J. (2022). Replica-Mold Nanopatterned PHEMA Hydrogel Surfaces for Ophthalmic Applications. Sci. Rep..

[98] Smith B.T., Belani S., Ho A.C. (2005). Ultraviolet and Near-Blue Light Effects on the Eye. Int. Ophthalmol. Clin..

[99] Mainster M.A. (1978). Spectral Transmittance of Intraocular Lenses and Retinal Damage from Intense Light Sources. Am. J. Ophthalmol..

[100] Henderson B.A., Grimes K.J. (2010). Blue-Blocking IOLs: A Complete Review of the Literature. Surv. Ophthalmol..

[101] Artigas J.M., Felipe A., Navea A., Artigas C., García-Domene M.C. (2011). Spectral Transmittance of Intraocular Lenses under Natural and Artificial Illumination. Ophthalmology.

[102] International Organization for Standardization, G.S. ISO 11979-3:2012; Ophthalmic Implants—Intraocular Lenses—Part 3: Mechanical Properties and Test Methods. https://www.iso.org/standard/55681.html.

[103] Remón L., Siedlecki D., Cabeza-Gil I., Calvo B. (2018). Influence of Material and Haptic Design on the Mechanical Stability of Intraocular Lenses by Means of Finite-Element Modeling. J. Biomed. Opt..

[104] Lane S., Collins S., Das K.K., Maass S., Thatthamla I., Schatz H., Van Noy S., Jain R. (2019). Evaluation of Intraocular Lens Mechanical Stability. J. Cataract Refract. Surg..

[105] Gu X., Chen X., Yang G., Wang W., Xiao W., Jin G., Wang L., Dai Y., Ruan X., Liu Z. (2020). Determinants of Intraocular Lens Tilt and Decentration after Cataract Surgery. Ann. Transl. Med..

[106] Bozukova D., Pagnoulle C., Jérôme C. (2013). Biomechanical and Optical Properties of 2 New Hydrophobic Platforms for Intraocular Lenses. J. Cataract Refract. Surg..

[107] Tortolano L., Mrad O., Manerlax K., Khalfoun C., Yousfi R., Saunier J., Secretan P., Yagoubi N. (2022). Comparative Stability of Intraocular Lenses during 2–20 Years of Artificial Aging, Potential Effects in Terms of Biocompatibility. J. Appl. Polym. Sci..

[108] Werner L., Storsberg J., Mauger O., Brasse K., Gerl R., Müller M., Tetz M. (2008). Unusual Pattern of Glistening Formation on a 3-Piece Hydrophobic Acrylic Intraocular Lens. J. Cataract Refract. Surg..

[109] International Organization for Standardization: Geneva, S. ISO 11979-5:2020; Ophthalmic Implants—Intraocular Lenses—Part 5: Biocompatibility. https://www.iso.org/standard/72602.html.

[110] Carraway J.W., Daniel E.M., Gilger B. (2013). Study Design and Methodologies for Study of Ocular Medical Devices. Ocular Pharmacology and Toxicology.

[111] Chehade M., Elder M.J. (1997). Intraocular Lens Materials and Styles: A Review. Aust. N. Z. J. Ophthalmol..

[112] Werner L. (2008). Biocompatibility of Intraocular Lens Materials. Curr. Opin. Ophthalmol..

[113] Werner L., Legeais J.-M., Nagel M.-D., Renard G. (1999). Neutral Red Assay of the Cytotoxicity of Fluorocarbon-Coated Polymethylmethacrylate Intraocular Lenses In Vitro. J. Biomed. Mater. Res..

[114] Kao E.C.Y., Seo J., McCanna D.J., Subbaraman L.N., Jones L.W. (2022). In Vitro Assessment of the Biocompatibility of Chemically Treated Silicone Materials with Human Lens Epithelial Cells. Sci. Rep..

[115] Tortolano L., Serrano C., Jubeli E., Saunier J., Yagoubi N. (2015). Interaction of Intraocular Lenses with Fibronectin and Human Lens Epithelial Cells: Effect of Chemical Composition and Aging. J. Biomed. Mater Res. A.

[116] Özyol P., Özyol E., Karel F. (2017). Biocompatibility of Intraocular Lenses. Turk. Oftalmol. Derg..

